# Chronic temporomandibular disorder pain patients with a history of neuropathic pain treatment: a narrative research on their diagnosis and treatment history

**DOI:** 10.1186/s12903-023-03796-0

**Published:** 2024-01-04

**Authors:** Jeanne H. M. Baggen, Anna C. Koevoets, Michail Koutris, Monique A. H. Steegers, Frank Lobbezoo

**Affiliations:** 1 Department of Orofacial Pain & Dysfunction, Academic Centre for Dentistry Amsterdam (ACTA), University of Amsterdam and Vrije Universiteit Amsterdam, Gustav Mahlerlaan, 3004, 1081 LA Amsterdam, The Netherlands; 2 Amsterdam UMC, Vrije Universiteit Amsterdam, Anesthesiology, Amsterdam, De Boelelaan 1117, Amsterdam, Netherlands

**Keywords:** TMD pain, Neuropathic pain, Chronic orofacial pain, Narrative research

## Abstract

The aims of this qualitative research were (1) to gain more insight in the diagnostic and treatment history of patients with chronic temporomandibular disorder (TMD) pain; (2) to get a deeper understanding of possible factors that are involved in the possible delay in setting a TMD-pain diagnosis and receiving appropriate treatment; and (3) to get a deeper understanding of the perspectives and experiences of chronic TMD-pain patients on the possible improvement of various aspects of their diagnostic and treatment journey.

**Methods**

In this narrative research, semi-structured interviews took place with patients who experienced chronic orofacial pain (OFP) for at least three years before getting diagnosed with, and treated for, TMD pain by an OFP specialist in an interprofessional setting.

**Results**

In total, ten patients were interviewed in-depth. Patients experienced their chronic OFP in different ways, but all reported a significant impact of their pain on their quality of life. All patients visited numerous health care professionals before their TMD diagnosis was set. Among others, they underwent anti-neuropathic pain medication therapies and invasive surgeries, which did not significantly reduce their chronic OFP. The interprofessional TMD-pain treatment reduced the suffering of the chronic OFP substantially, also 6 months after the start of therapy, and improved the quality of life for all patients. In most of them, the OFP intensity was also decreased.

**Conclusion**

Chronic TMD-pain patients with a history of neuropathic pain treatment may experience a long journey until receiving the appropriate diagnosis and treatment. This stresses the need to improve the implementation of chronic TMD-pain guidelines.

**Supplementary Information:**

The online version contains supplementary material available at 10.1186/s12903-023-03796-0.

## Introduction

The prevalence of Chronic orofacial pain (OFP) in the general adult population is around 13% (range 1–48%) [1]. OFP has substantial impact on the quality of life of its sufferers [2]. Recently, a collaborative work on the classification of OFP conditions has been published (International Classification of Orofacial Pain; ICOP) [3]. This classification recognizes the following diagnostic categories: OFP attributed to disorders of dento-alveolar and anatomically related structures, myofascial OFP, temporomandibular joint (TMJ) pain, orofacial neuropathic pain, OFPs resembling presentations of primary headaches, and idiopathic OFP. The last chapter of the ICOP is devoted to the importance of a psychosocial assessment of OFP patients in the diagnostic process [3].

From all OFP entities included in the ICOP, temporomandibular disorder (TMD) pain is considered the second most common cause for OFP, after dental pain. TMD pain is an umbrella term referring to pain of musculoskeletal origin in the orofacial region [4]. Its prevalence is approximately 10% in the general adult population and presents at least two times more often in women than in men, peaking in age group 20–40 years [5, 6]. TMD pain has a mild to moderate intensity and a fluctuating nature. Its aetiology includes biological, psychological, and social factors [7–9]. The dynamic interaction among these factors can cause TMD pain to become chronic, being accompanied by aggravation of existing pain complaints [10, 11]. For TMD diagnosis, Diagnostic Criteria for TMD (DC/TMD) are internationally accepted for both clinical and research use [12]. The DC/TMD assesses physical (Axis I) and psychosocial (Axis II) characteristics [12]. The DC/TMD is fully incorporated in the ICOP.

TMD pain is considered nociceptive pain [13]. However, some TMD patients seem to experience an altered nociception that can resemble clinical symptoms of neuropathic pain [14–16]. Nociplastic pain is, according to the International Association for the Study of Pain, defined as a type of pain just like nociceptive and neuropathic pain [17]. In some TMD patients, nociplastic pain may be a better descriptor of the clinical appearance of altered nociception [13]. Furthermore, patients can suffer simultaneously from a combination of nociceptive and neuropathic pain, yielding a complex clinical appearance [3]. Consequently, this may lead to difficulties determining the appropriate diagnosis and treatment approach. In turn, this may result in applying unnecessary invasive treatments [18, 19], while TMD pain is commonly managed non-invasively [8, 20, 21]. This all illustrates that the complexity of TMD pain may cause a significant delay in setting correct diagnoses and, in case of an incorrect diagnosis, may lead to unnecessary invasive treatments [22–24]. To prevent this, guidelines have been published internationally [4, 25]. The implementation of these guidelines, however, is unclear. To that end, narrative research on patients’ perspectives and experiences is needed.

Therefore, our primary aim was to gain more insight in the diagnostic and treatment history of patients with chronic TMD pain. It was hypothesized that patients with such pain often have a long history of unsuccessful treatments. The secondary aim was to get a deeper understanding of possible factors that are involved in possible delay in setting a TMD-pain diagnosis and receiving appropriate treatment. The hypothesis was that professionals are not always aware of, or do not follow, the existing guidelines [4, 25]. The tertiary aim was to get a deeper understanding of the perspectives and experiences of chronic TMD-pain patients on the possible improvement of various aspects of their diagnostic and treatment journey.

## Methods

### Study design and patients

The design of this study was a narrative research, based on semi-structured in-depth interviews with patients. The research population included adults with chronic orofacial pain (OFP) that lasted longer than three years before a TMD-pain diagnosis was set according to the Diagnostic Criteria for Temporomandibular Disorders (DC/TMD) [12]. All included patients had already received treatment suitable for neuropathic pain, like pharmaceutical therapy or surgeries performed by a medical specialist or general practitioner. Some patients also took medication for pain in other locations of their body. Although some patients reported that their medication was not helpful, they still used it at the time of the intake. Patients who were using pain medication were advised not to stop during the TMD-treatment path. At the end of the treatment, we advised the patients to contact their specialist/general practitioner to reduce the pain medication slowly, if possible. After setting the TMD-pain diagnosis, all patients received interprofessional treatment [26], including splint therapy, orofacial physiotherapy, psychotherapy, and in some cases speech therapy, in the same period. In order to be included in the study, patients had to report subjective substantial pain relief during the last evaluation appointment for their TMD pain, approximately six months after the start of TMD-pain therapy. After the TMD-pain treatment, most patients believed that the TMD pain was the main cause of their orofacial pain and that the neuropathic pain treatments were unsuccessful. The inclusion and exclusion criteria are shown in Table 1. Patients from two different clinics were approached and included in this study, namely the clinic of the Department of Orofacial Pain and Dysfunction of the Academic Centre for Dentistry Amsterdam (ACTA), the Netherlands, and a referral clinic for OFP patients in Nijmegen, the Netherlands. An OFP specialist, J.B., identified potentially eligible patients, set the TMD-pain diagnosis, and treated all participating patients in the past. The selection process of the eligible patients was based on the inclusion and exclusion criteria. Patient age, gender, ethnicity, and other demographic information was not considered for the selection. Eligible patients were approached by phone. Patients who agreed to participate received an Informed Consent and Research Information sheet by e-mail. The patients signed the informed consent and sent it back to the OFP specialist. The number of included patients was determined based on saturation of data. Data was saturated when important constituencies were sufficiently presented to obtain sound qualitative evidence and when the point of diminishing return was reached where increasing the sample size would no longer contribute to the evidence [27].

**Table 1:** Inclusion and exclusion criteria

**Inclusion criteria**
- ≥ 18 years of age
- Chronic OFP ≥ 3 years before a TMD-pain diagnosis was set by an OFP specialist
- TMD-pain diagnosis was set according to the DC/TMD
- Pharmaceutical therapy or surgeries as neuropathic pain treatment in the past
- Interprofessional TMD-pain treatment (splint therapy, orofacial physiotherapy, and psychotherapy)
- Subjective substantial TMD-pain relief during the last evaluation appointment 6 months after the start of interprofessional TMD-pain therapy
**Exclusion criteria**
- TMD-pain diagnosis was not set by the DC/TMD

*OFP* Orofacial Pain, *TMD* Temporomandibular Disorders, *DC/TMD* Diagnostic Criteria for Temporomandibular Disorders

### Informed consent and ethical approval

The study protocol was reviewed and approved by the Medical-Ethical Review Committee of the VUmc (METc VUmc) (file 2019.108). An amendment (approved by the Ethical Committee Academic Centre for Dentistry Amsterdam (ACTA-ETC); file 202082) allowed the inclusion of patients from a referral clinic for OFP patients in Nijmegen as well as performing the interviews online due to the COVID-19 crisis, which prevented the original plan for live interviews.

### Interview procedure and topic guide

he interview took place through video conference and was audio-recorded in digital format, professionally transcribed verbatim, and then checked for accuracy against the original recordings. The interviewer, J.B., was an OFP specialist with 9 years of experience who had no prior experience in performing qualitative interviews for research purposes. J. B. set the TMD-pain diagnosis and treated all participating patients in the past. The semi-structured in-depth interviews were based on a topic guide, which included key subjects that are shown in Table 2 [27]. When an extra topic was added by a patient, the topic guide was adjusted. The final topic guide after adjustments is given in Additional file 1; the additions are italicized.

**Table 2:** Main topics in the topic guide; details in Additional file 1

1. Background of the patient
2. Course of OFP complaints before TMD-pain treatment
3. Medical and dental treatment history before TMD-pain treatment
4. Interprofessional treatment of TMD pain
5. Current OFP condition

*OFP* Orofacial Pain, *TMD* Temporomandibular Disorders

### Thematic qualitative analysis

Data was analysed using the thematic qualitative analysis approach [27]. This was performed by two researchers (J.B. and A.K.) parallel to the interviews. The preconceptions were written down as main topics in Additional file 1 before the start of the study, while the italicized text in the appendix represents additions to the topic guide based on insights that were obtained during the interviews. The transcribed text from the interviews was all put in one document. Based on the data, themes and subthemes were identified to construct an initial framework and to accommodate the experiences of the patients. The thematic framework was used to organize the data based on the interviews.

## Results

### Patients

Eighteen patients were approached for participation. Eleven patients fulfilled the inclusion criteria and were willing to participate in the study. Seven patients were excluded because: they had chronic OFP shorter than three years before the TMD pain diagnosis was set (*n* = 3), did not receive pharmaceutical therapy or surgeries as neuropathic pain treatment in the past (*n* = 2), did not want to sign the informed consent (*n* = 1) or cancelled because of COVID-19 (*n* = 1). For patients who did not remember the name of the anti-neuropathic pain medication they had taken in the past, a general identifier “anti-neuropathic pain medication” was used. After interviewing ten patients, the data was saturated, i.e., there were no new topics after nine interviews. Hence, the eleventh patient was not interviewed due to saturation of data [27]. The study sample characteristics are shown in Table 3.

**Table 3:** Patient characteristics

	**Gender (male/female)**	**Age at time of the interview (years)**	**Years of OFP before intake with OFP specialist**	**TMD-pain diagnoses**	**Click TMJ**
**Patient 1**	Male	71	20	Myalgia (m. masseter right side)	-
**Patient 2**	Female	43	4	Myalgia (m. masseter left side)	-
**Patient 3**	Female	48	3	Myalgia	Click right and left side
**Patient 4**	Female	62	10	Myalgia (m. masseter right side, m. temporalis right side)	-
**Patient 5**	Female	59	10	Myofascial pain with referred pain (m. masseter right side)	-
**Patient 6**	Male	59	3	Myalgia (m. masseter left side), arthralgia (left side)	Click right and left side
**Patient 7**	Female	38	16	Myalgia (m. masseter right and left side)	-
**Patient 8**	Female	54	3	Myalgia (left side)	Click right side
**Patient 9**	Female	71	7	Myofascial pain with referred pain (left side)	-
**Patient 10**	Male	60	40	Myalgia (m. masseter right and left side)	Click on the right side

*OFP* Orofacial Pain, *TMD* Temporomandibular Disorders

### Case description per patient

#### Patient #1

Male, age 71, referred by the general practitioner. The history of caregivers was a dentist, endodontist, general practitioner, and ENT specialist. The history of treatment was anti-neuropathic pain medication. Patient had 20 years of OFP before the intake. Comorbid heart disease and bypass surgery. The TMD-pain diagnosis was myalgia in the masseter muscle, right side. In the biopsychosocial (BPS) history taking, the patient indicated that he had worked for 60 h per week during his working life and had worries about the health of his ill wife. Patient felt hopeless about the OFP complaint and felt he had to accept it as part of his life.

#### Patient #2

Female, age 43, referred by the neurosurgeon. The history of caregivers was a dentist, general practitioner, neurosurgeon, rheumatologist, and neurologist. The history of treatment was pregabalin and etoricoxib, as well as a Janetta procedure in the past. Patient had 4 years of OFP before the intake. Comorbid rheumatoid arthritis since age 28, possible diagnosis of scleroderma, and possible Transcient Ischemic Attack. The TMD-pain diagnosis was myalgia in the masseter muscle, left side. BPS history taking showed that the patient has difficulty naming stress factors in her life. After the TMD treatment, the patient and general practitioner started to diminish the pain medication.

#### Patient #3

Female, age 48, referred by the dentist. The history of caregivers was a dentist, periodontist, oral surgeon, general practitioner, and ENT specialist. The history of treatment was anti-neuropathic pain medication. Patient indicates that she used to take a cocktail of medication in the past and went through life “stoned”. Patient had 3 years of OFP before the intake. Comorbid chronic headache. The TMD-pain diagnosis was myalgia and TMJ with a click on the left and right side. BPS history taking shows that she is very sensitive and has a hard time letting things go. She has a history of cancer in the family, including her son, and patient feared that the OFP complaint was due to cancer. The patient did not use medication at the time of the intake, nor at the end of the TMD treatment.

#### Patient #4

Female, age 62, referred by the ENT specialist. The history of caregivers was a dentist, periodontist, implantologist, general practitioner, neurologist, and neurosurgeon. The history of treatment was oxcarbazepine, and a Janetta procedure was scheduled but was not performed. Patient had 10 years of OFP before the intake. Comorbid high blood pressure. The TMD-pain diagnosis was myalgia in the masseter muscle, right side, as well as in the temporalis muscle, right side. BPS history taking shows that the patient has high mental pressure from her executive function at a hospital, as well as from her husband losing his job. Has a high sense of responsibility and finds it difficult to take necessary breaks. Patient had no more complaints after TMD treatment, used oxcarbazepine during the intake, but stopped using this medication by herself during the treatment path despite the advice not to.

#### Patient #5

Female, age 59, referred by the neurosurgeon. The history of caregivers was a dentist, general practitioner, neurologist, and neurosurgeon. The history of treatment was oxcarbazepine and a Janetta procedure. A Motor Cortex Stimulation was scheduled but not performed. Patient had OFP for 10 years before the intake. Comorbid high blood pressure and kidney stones. The TMD-pain diagnosis was myofascial pain with referred pain in the masseter muscle, right side. BPS history taking shows that the patient worries about her grandchildren a lot and fears that they will get sick. Patient had suicidal ideation once because of the OFP but pushed through despite of her suffering. Patient no longer had complaints at the end of the treatment but felt very sad that nobody had taught her how to relax her jaws for so long, which had caused her to be unable to function socially. Patient used oxcarbazepine during the intake and is going to reduce its usage with the general practitioner.

#### Patient #6

Male, age 59, referred by the pain specialist. The history of caregivers was a dentist, pain specialist, general practitioner, psychologist, physiotherapist, orofacial physiotherapist, chiropractor, and neurologist. The history of treatment was amitriptyline, brufen, paracetamol, pregabalin, a facet blockage, and an occlusal splint. Physiotherapy was performed prior to the intake. Patient had OFP for 3 years before the intake. The TMD-pain diagnosis was myalgia in the masseter muscle, left side, arthralgia on the left side, and TMJ clicking on the left and right side. BPS history taking showed that the dentist had referred the patient several times for TMD to an OFP specialist. The general practitioner did not believe that there was a musculoskeletal cause for the complaints, which caused the patient to also disregard it, even though he received a diagnosis of trigeminus neuralgia from the general practitioner. Invasive neuropathic pain treatment did not help, after which the patient wanted to try TMD treatment.

#### Patient #7

Female, age 38, referred by the neurosurgeon. In the referral letter, the surgeon reports: “The MRI shows no neurovascular conflicts, however, there is a possibility of a nociceptive causal factor for the orofacial pain”. The history of caregivers was a general practitioner, neurologist, and neurosurgeon. The history of treatment was carbamazepine, gabapentin, a Janetta procedure, as well as tooth extractions. Both the medication and Janetta procedure did not help. Patient had OFP for 16 years before the intake. The TMD-pain diagnosis was myalgia in the masseter muscle, right side. BPS history taking shows that the patient was very self-conscious in primary and secondary school. Came out of the closet at 17 years old, at which point the orofacial pain started.

#### Patient #8

Female, age 54, referred by the general practitioner. The history of caregivers was a general practitioner and ENT specialist. The history of treatment was anti-neuropathic pain medication and anti-fungal ointment. The patient had OFP for 3 years before the intake. The TMD-pain diagnosis was myalgia on the left side, with TMJ clicking on the right side. BPS history taking shows a divorcee who cares for two children. A high amount of tension and stress between her and her children. Had been a caretaker for both of her parents who were sick. Does not want to continue living with her mouth complaints but says she does not give up easily.

#### Patient #9

Female, age 71, referred by the pain specialist. The history of caregivers was a dentist, general practitioner, neurologist, neurosurgeon, and pain specialist. The history of treatment was carbamazepine, lacosamide, an occlusal splint and occlusion analysis, two Sweet procedures, and a Janetta procedure. The patient had OFP for 7 years before the intake. Comorbid unknown sleeping disorder. The TMD-pain diagnosis was myofascial pain with referred pain on the left side. BPS history taking shows a family dynamic with an authoritarian and abusive fatherly figure. Physical abuse was commonplace in her youth. Patient is the eldest daughter of 8 children, with a background from the Dutch East Indies, and the patient had to act as the secondary mother to the family. High demands from the family were placed on the daughter. Family matters were not allowed to be discussed. Patient views herself as perfectionistic and is angered and stressed if things don’t go perfectly. Has a high urge to perform and is always busy, with no internal rest. Did not want to visit the psychologist at first.

#### Patient #10

Male, age 60, referred by the dentist. The history of caregivers was a dentist, general practitioner, ENT specialist, psychologist, and psychiatrist. The history of treatment was anti-neuropathic pain medication and a splint. The patient had OFP his entire life. Comorbid headache, ADD, sleep apnea with a gastric bypass, and loss of overweight. The TMD-pain diagnosis was myalgia in the masseter muscle, right and left side, with TMJ clicking, right side. BPS history taking shows the father frequently abused the patient sexually when the patient was younger, which the patient kept quiet for a long time. During the intake, the patient’s father was dying. The patient wanted to discuss the abuse with his father before he passed away. The father did not want to discuss what happened and the family judged the patient instead. Consequently, the patient is still using the same medication as during the intake. He says that what has been built up over 50 years needs more time to heal.

### Thematic qualitative analysis

The thematic qualitative analysis was performed parallel to the ten interviews. The themes and subthemes which arose based on the obtained data constituted the thematic framework (Table 4). Below, all themes will be described separately, including some important patient quotes. The specific patient is indicated with a number sign (#). Extra quotes are included in Additional file 2 and will be referred to with a number sign (#) referring to the specific patient as well**.**

**Table 4:** Thematic framework; themes and subthemes on basis of the analysis

**Theme 1:** Chronic OFP complaints before TMD-pain treatment
1. OFP complaints and course
2. Impact on daily life, work, other activities, and sleep
3. Patient’s own idea about the cause of chronic OFP
**Theme 2:** History of treatments of chronic OFP before TMD-pain treatment
1. Medical treatments’ history
2. Dental treatments’ history
**Theme 3:** Experiences with TMD-pain treatment
1. Experience of getting diagnosed with TMD pain
2. Experience with interprofessional team
3. Experience with OFP specialist
4. Experience with the occlusal splint
5. Experience with orofacial physiotherapist
6. Experience with speech therapist
7. Experience with psychologist
**Theme 4:** Bruxism and other oral behaviours
1. Bruxism and other oral behaviours before TMD-pain treatment
2. Bruxism and other oral behaviours during TMD-pain treatment
3. Bruxism and other oral behaviours after TMD-pain treatment
**Theme 5:** Chronic OFP complaints after TMD-pain treatment
1. Start of long-lasting pain reduction
2. OFP reduction
3. Current pain medication
4. Thoughts of patients about the cause of chronic OFP after TMD-pain treatment
**Theme 6:** Patient’s perspective on improving chronic OFP care
1. OFP knowledge among medical and dental professionals
2. History of invasive treatments for OFP
3. Interprofessional team

*OFP* Orofacial Pain, *TMD* Temporomandibular Disorders

## Theme 1: Chronic OFP complaints before TMD-pain treatment

### OFP complaints and course

The OFP characteristics were different for each patient, with complaints and course being reported as totally atypical phenomena. Patients #4, #5, #6, and #7 felt a burning or electric-shock-like pain (see quotes Additional file 2 #1.1.1). Some of the patients experienced a feeling of inflammation or tension in the teeth and/or gums. Patient #8 described the OFP as a nettle on the tongue. Others felt a stabbing pain in the ear, nose, eye, or cheek area (#1.1.2). Patient #1 described his OFP as a blocked nose. Headache was also a frequently reported symptom. The course of the OFP complaints differed from a constant to a fluctuating pain. Sometimes, the OFP was only present on one side.

### Impact on daily life, work, other activities and sleep

All patients mentioned a high negative impact of their OFP on their daily life (#1.2.1). OFP impeded daily activities for patients, and patients did not feel any social support from their family and friends: their social environment misunderstood their OFP complaints, which caused social isolation. Patient #3 compared her OFP complaints with the contagious disease tuberculosis, which she had when she was 28 years old; she experienced loneliness very deeply (#1.2.2). Patients #2, #5, and #8 name OFP a “suicide” disease (#1.2.3). Waking up because of OFP was a common complaint among the patients (#1.2.4). The disturbed sleep quality resulted in even more pain and less energy for daily activities, like going to work and seeing friends (#1.2.5).

### Patients’ own idea about the cause of chronic OFP

By some patients, the cause of chronic OFP was considered unknown for all those years. All of them considered that their OFP complaints were due to a physical cause. Patient #3 thought that whitening her teeth by the dentist caused her OFP, because this was the moment the OFP started. In the case of patient #4, OFP started after being treated with dental implants (#1.3.1). Family members and friends often had an opinion about the cause of OFP as well. *“Everyone always told me that stress was the cause of OFP, but I didn’t believe that at first, I just did my thing”* (#7). Patient #5 had the idea that muscle tension was the cause of OFP.

## Theme 2: History of treatments of chronic OFP before TMD pain treatment

Patients got referred to the OFP specialist by different health-care professionals: general practitioners, dentists, ENT doctors, neurosurgeons, and anaesthesiologists. Most of them visited a wide variety of health care professionals in the dental and medical field, looking for pain relief, before they were referred to the OFP specialist (#2.0.1). The accumulation of referrals is shown in Table 5. Per patient, a lot of different treatments were given for OFP reduction, see Table 6.

**Table 5:** Overview of health-care providers visited by the included patients for OFP relief before the TMD-pain diagnosis was set

Patient	Dental sector	Medical	Alternative sector	Referrer to OFP specialist
Patient 1	Dentist Endodontist	General practitioner ENT specialist		General practitioner
Patient 2	Dentist	General practitioner Neurosurgeon Rheumatologist Neurologist		Neurosurgeon
Patient 3	Dentist Periodontist Oral surgeon	General practitioner ENT specialist		Dentist
Patient 4	Dentist Periodontist Implantologist	General practitioner Neurologist Neurosurgeon		ENT specialist
Patient 5	Dentist	General practitioner Neurologist Neurosurgeon		Neurosurgeon
Patient 6	Dentist	General Practitioner Psychologist Physiotherapist Orofacial physiotherapist Chiropractor Neurologist		Dentist & Anaesthesiologist – pain specialist
Patient 7	Dentist Centre for special dentistry	General practitioner Neurologist Neurosurgeon		Neurosurgeon
Patient 8	Dentist Oral surgeon	General practitioner ENT specialist	Chinese acupuncturist Organic food centre	General practitioner
Patient 9	Dentist Prosthodontist and restorative dentist	General practitioner Neurologist Neurosurgeon Anaesthesiologist – pain specialist		Anaesthesiologist – pain specialist
Patient 10	Dentist	General practitioner ENT specialist Psychologist Psychiatrist	Naturopath	Dentist

*OFP* Orofacial Pain, *TMD* Temporomandibular Disorder, *ENT* Ear Nose Throat

**Table 6:** Overview of performed or scheduled dental and medical treatments for OFP reduction of the included patients. All medication which is not specified, is due to the patients not remembering the name of their medication

Patient	Medication	Surgery	Other
Patient 1	Anti-neuropathic pain medication		
Patient 2	Pregabalin Etoricoxib	Janetta procedure (2017)	
Patient 3	Anti-neuropathic pain medication		
Patient 4	Oxcarbazepine	Scheduled, but not performed Janetta procedure (2017)	
Patient 5	Oxcarbazepine	Janetta Procedure (2017) Scheduled, but not performed Motor Cortex Stimulation	
Patient 6	Amitriptyline Brufen Paracetamol Pregabalin	Facet blockage (2017)	Splint Physiotherapy
Patient 7	Carbamazepine Gabapentin	Janetta procedure (2016)	Tooth extractions
Patient 8	Anti-neuropathic pain medication Anti-fungal ointment		
Patient 9	Carbamazepine Lacosamide	Sweet procedure (2012) Sweet procedure (2015) Janetta procedure (2016)	Splint Occlusion analysis with T-scan
Patient 10	Anti-neuropathic pain medication		Splint

*OFP* Orofacial Pain

### Medical treatments’ history

All included patients had a long medical history. Most patients got diagnosed with trigeminal neuralgia in the past (#2.1.1).

Different medications were prescribed to patients. Patient #8 would not use anti-neuropathic pain medication anymore, but only paracetamol as a pain killer, which did not help either. Other patients had tried out all kinds of medication; anti-epileptic drugs, antidepressants, and morphine. Patient #2 started out with paracetamol for her predominant OFP, before she tried taking amitriptyline, pregabalin, and carbamazepine. No medication reduced her OFP complaints (#2.1.3). Patient #9 used lacosamide, an anti-epileptic drug which worked best for her. Oxcarbazepine was the medication of choice of the doctors of patients #4 and #5, which helped a bit for patient #5. Gabapentin, tramadol, and oxycodone were other medications that were prescribed as reported by the patients (#2.1.4). The pain medication caused side effects in patients. Carbamazepine induced skin rash over the entire body of patient #9. Patient #6 struggled with hypotension due to amitriptyline (#2.1.5).

About half of the patients had undergone invasive surgeries, see Table 5. Patient #2 underwent a Janetta procedure, because according to the neurosurgeon a blood vessel was too close to a nerve. In the beginning, the procedure seemed to help reduce OFP, but after a check-up with the dentist, the same pain came back (#2.1.6). The Janetta procedure did not help at all with patient #5. *“I have had the Janetta procedure. In retrospect, I think that the operation should not have been performed. Luckily, there were no complications”* (#5). Advised by her sister, patient #7 was referred to the OFP specialist after the neurosurgeon proposed another surgery, when the OFP relapsed one year after the first Janetta procedure. Due to the seasonal fluctuation of her OFP, she thought the Janetta procedure seemed to help at first. (# 2.1.7). Patient #7 had headache for weeks after the Janetta procedure. Patient #9 underwent the Sweet procedure, which resulted in immediate OFP reduction. Unfortunately, the OFP of patient #9 came back again after three years. She then underwent a second Sweet procedure. This time, her OFP disappeared for three months. Patient #9 eventually underwent the Janetta procedure, after which she spent three weeks in the hospital to recover from this third operation (#2.1.8).

### Dental treatments’ history

Most dentists did not know how to deal with the OFP complaints of their patients (#2.2.1). Some dentists provided patients with an occlusal splint, but that did not help to reduce the OFP complaints (#2.2.2). The dentist of patient #10 kept on selectively grinding his molars until the patient no longer felt pressure in his teeth. The pain in the teeth remained every time the dentist grinded a molar (#2.2.3). Dental problems were common among patients; toothaches of unknown aetiology, root canal treatments, tooth extractions, fractured teeth, and broken restorations. Patient #6 did not believe the dentist when she told him the OFP was the result of bruxism. That there were no signs of oral pathologies found on X-rays did not convince him. The teeth of patients #7 and #9 were extracted while this was redundant (#2.2.4).

## Theme 3: Experience with TMD-pain treatment

### Experience of getting diagnosed with TMD pain

TMD-pain diagnoses were set according to the DC/TMD, with data from the Diagnostic Questionnaire, a clinical BPS history taking and examination. The relationship between TMD pain, bruxism, and psychological distress was explained to the patients. *“Well, I thought, okay, is it so simple?”* (#2). Patients were relieved when diagnosed with TMD pain (#3.1.1). The switch from a somatic approach of previous practitioners to a BPS approach, including a clinical BPS history taking by the OFP specialist, was confronting for most patients (#3.1.2). Patients did not realize how much stress they were carrying with them through life until the first visit at the OFP specialist. *“In the beginning, I had to think about it, because I never had the feeling that OFP was connected to stress. I was just doing my thing, but apparently my bucket was just full at some point”* (#7).

### Experience with interprofessional team

The OFP specialist diagnosed patients with TMD pain and referred all patients to an orofacial physiotherapist, a psychologist, and sometimes to a speech therapist. Simultaneous care from each profession in an interprofessional team and joint consultations of the team members to discuss their individual patient approach to TMD-pain treatment are characteristics of this care. The interprofessional treatment was an intense period for patients. *“I found it [interprofessional treatment] very intensive, but I experienced it as pleasant. It [different disciplines in interprofessional treatment] logically came together”* (#3). Most of the patients had a good experience with the BPS approach of members of the interprofessional team (#3.2.1).

### Experience with OFP specialist

In order to treat chronic OFP, addressing potential pain- and non-pain-related stressors are essential. Importantly, the OFP specialist identified the potential stressors and maladaptive coping skills, according to a clinical BPS history taking during initial screening and setting the TMD-pain diagnosis. Furthermore, the patient was educated about the role of stress in the chronic pain experience of the patient. Afterwards, when the patient understood the underlaying mechanism of stress and pain experience, the OFP specialist referred the patient to the most suitable mental health clinician, for example a psychologist. The OFP specialist made a splint for the patient and was counselling the patient in order to become aware of bruxism or other oral behaviours and how the understanding of the BPS approach is developing by the patient.

The explanation of the cause of OFP was important to hear for the patients. *“The first step was teaching me to accept that I was a biter. The OFP specialist was very clear about this every time; ‘you are a biter; you bite everything to pieces.’ For me the specialist’s perseverance was necessary in order to accept that this was, and still is, the problem”* (#6). All patients found it really important that there finally was a practitioner who really listened to their problem and their story (#3.3.1). Patient #5 learned from the OFP specialist that she had to take better care of herself. Patients learned that certain stressful life events can be associated with OFP and that there is a connection between mind and body. Patient #10 learned how underlying stress factors, for example sexual abuse, can be related with TMD pain.

### Experience with the occlusal splint

A custom-made occlusal splint for the lower or upper jaw was given to patients by the OFP specialist. It was explained that this is a tool to get aware of bruxism and other oral behaviours during wakefulness, and that it is a protection against damage of their dentition due to grinding or clenching during sleep (#3.4.1). Most of the patients cannot sleep anymore without the occlusal splint (#3.4.2). Only patient #4 was not happy with the occlusal splint; she reported it hurt when she took it out in the morning. Some patients recognized that only splint therapy would not have alleviated their OFP complaints (#3.4.3). Patient #10 described being more aware of bruxism while wearing the occlusal splint during the day (#3.4.4).

### Experience with orofacial physiotherapist

The orofacial physiotherapist taught patients how to relax their jaw and how to massage their masticatory muscles and gave the patients an exercise programme for muscle relaxation to be performed at home. *“I really learned there [with the physiotherapist] how to relax my jaw, the physiotherapist was my muse”* (#5). Patients learned how it feels to relax their masticatory muscles. Most of them were not even familiar anymore with the feeling of relaxation (#3.5.1).

### Experience with speech therapist

The speech therapist taught patients what a relaxed position of the tongue is and instructed them exercises to practice at home. *“I always put my tongue against my lower jaw. Now I put it up against my palate, this seems to be natural for everyone, but apparently for me it was not. I pressed my tongue very hard against my lower jaw, especially when I was working”* (#3). Patients #5 and #8 also went to the speech therapist and learned how to relax their tongues.

So, three of ten patients were referred to a speech therapist, because it was expected they would benefit from this kind of treatment (#3.6.1).

### Experience with psychologist

The psychologist got a report from the clinical BPS history taking of the OPD specialist and assessed further stress factors and coping factors which could be related to TMD pain.

The first time when some patients heard they were referred to a psychologist, they did not feel taken seriously, thought it was nonsense (#3.7.1), or were not used to the BPS approach (#3.7.2). *“I found it very difficult to be referred to a psychologist – I mean, I wasn’t cracking up or anything!”* (#1). Patients #4, #5, #7, and #8 found the referral to a psychologist confrontational. *“You go for the treatment, and then you go for it 100%. This consultation with the psychologist is part of the treatment. Afterwards, I thought it would be good for everyone to talk to a psychologist at least once”* (#4). Some of the patients did not think they would be open to psychotherapy, but they changed their opinion after visiting the psychologist (# 3.7.3). After the appointments with the psychologist, all patients, except for one, valued the treatment (#3.7.4).

The patients learned more about their personality, pitfalls, and stress factors, and learned how to set boundaries (#3.7.5). Patient #6 learned from the psychologist that he is a Highly Sensitive Person (HSP). During three sessions, he was taught how to deal with this (#3.7.6). Patients #4 and #7 learned from the psychologist to let go of ruminating thoughts about how people think of them, which caused them stress (#3.7.7). When patient #7 came out for the first time, she experienced a lot of stress (#3.7.8). Many patients describe themselves as a brooder, the type of person who keeps everything locked up (#3.7.9). Patient #10 struggled through life after he and his brothers were sexually abused by their father when they were kids. The realization that this sexual abuse could possibly be related with his TMD pain shocked him (#3.7.10). Other stressful events in his life were his burnout, his divorce, and the suicide attempt of his child (#3.7.11).

## Theme 4: Bruxism and other oral behaviours

### Bruxism and other oral behaviours before TMD-pain treatment

Almost all patients reported not being aware of bruxism or other oral behaviours during wakefulness or sleep before their TMD-pain treatment. Some patients said they started to notice oral behaviours and paid more attention to them after filling in the Diagnostic Questionnaire. Only patient #5 felt tension of her masticatory muscles, and patient #10 had the idea bruxism was present during sleep.

### Bruxism and other oral behaviours during TMD-pain treatment

The OFP specialist advised patients about bruxism activity and other oral behaviours during the first visit at the OFP clinic (#4.2.1). Patients described that after the first visit at the OFP specialist (s) he tried to increase awareness about bruxism. *“When I was in the car after the first visit, I realised that I was clenching my teeth. It was funny to find out that was a habit of mine”* (#2). Patients described that the first step was recognizing their oral habits, and then to work on decreasing the intensity and frequency of those habits (#4.2.2). Quitting oral habits took effort for most patients (#4.2.3). Patient #5 learned from the physiotherapist what she could change and improve. *“My jaw always felt tired. I always knew I was doing something wrong, but now I finally knew what it was. I learned how to correct this”* (#5).

### Bruxism and other oral behaviours after TMD-pain treatment

All patients became aware of bruxism and other oral behaviour at the end of the TMD-pain treatment. *“One of my children tried to commit suicide recently. Then I noticed the increase of stress in my jaw area again, I have to stay in control when that happens. I am now more aware of when I am grinding my teeth”* (#10).

## Theme 5: Chronic OFP complaints after TMD-pain treatment

### Start of long-lasting pain reduction

All patients mentioned that the fact that they started being treated by the interprofessional team was the starting point of the OFP reduction. Only patient #9 mentioned the medication she got from the anaesthesiologist as an important factor in OFP relief as well.

### Οrofacial pain reduction

Patients #3 and #7 had an OFP reduction of almost 100% at the end of the TMD-pain treatment. Patients #2, #4, #6, #8, and #9 had an OFP reduction of 50–70%. *“The OFP does not bother me very much anymore, unless something happens, or when I have a few nights of bad sleep. I recognize the influence this [psychological distress and bad sleep] has on the [orofacial] pain”* (#6).

All of the patients experienced TMD pain as a fluctuating pain. In work, stress, or concentrating moments there was an onset of the OFP. Some of the patients started using the tips and tricks they learned from the interprofessional team when there was pain present. Others forgot about the exercises after some time (#5.2.1). Patient #6 found it difficult to quantify the pain reduction during the interview. Patient #10 reported pain reduction at the end of TMD-pain treatment, but during the interview he couldn’t remember this anymore. He especially mentioned the increase of quality of life as a result of the TMD-pain treatment.

### Current pain medication

Patient #2 did not use any medication for OFP anymore, even though she used pregabalin, amitriptyline, and carbamazepine in the past. Patient #4 used carbamazepine only when it is really necessary. Patients #5 and #7 tried to reduce the dose of anti-neuropathic pain medication, but that has not yet been successful. *“I use carbamazepine and it is very hard for me to reduce the dose. When I try that, I get other nerve complaints instead, such as stabs of neuropathic pain in my left hip”* (#7). Patient #9 succeeded in diminishing 60% of the daily dose of lacosamide.

### Thoughts of patients about the cause of chronic OFP after TMD-pain treatment

All patients thought that psychosocial distress and tension on the masticatory system is related with their chronic OFP. Patient #10 believed in the relationship as well, but because he is still in pain, he finds the relation between bruxism and OFP difficult to accept, although he does understand that the diminishing of pain is an ongoing process. *“I am convinced that what has built up over almost 55 years of certain behaviour and physical symptoms will not just disappear after one-and-a-half years”* (#10). According to all these patients, medical specialists and general practitioners are often not familiar with chronic TMD pain (#2.1.2).

## Theme 6: Patients’ perspective on improving chronic OFP care

### Patients’ thoughts about OFP knowledge among medical and dental professionals

All patients experienced OFP for years and expressed their deep hope to improve general knowledge of chronic TMD pain among medical and dental professionals. *“I think many people could benefit from greater knowledge about this TMD pain, which will result in faster referral to the proper specialist. I had this [OFP] for three years; I wish I could have avoided it!”* (#3). Referring to the proper practitioner seemed to be the problem according to patients. *“I found it a shame that general practitioners and pain specialists are not aware they can refer someone with OFP to an OFP specialist”* (#9). Patient #8 even called several specialists he visited for OFP in the past to inform them. *“I called some practitioners who treated me in the past to say that if a similar case comes along they should damn well refer it to an OFP specialist”* (#8). Previous practitioners of patients lacked knowledge of bruxism (#6.1.1). Several patients are of the opinion their dentist should know about bruxism and other oral behavior and what consequences they can have. *“A dentist needs to know about tongue-pressing, and how it can affect OFP. That should be standard knowledge”* (#3). Patient #6 was the only patient who was referred to an OFP specialist before, at least twice in the past years. His dentist could not convince him of the possibility that bruxism was the cause of his OFP before. He got referred to the neurologist and neurosurgeon by his general practitioner. The medication and operation did not help for OFP reduction in patient #6. The next stop was an anesthesiologist, who was the third practitioner who referred patient #6 to the OFP specialist. *“I Didn’t see that the team could solve my problem, so it was difficult – I was sure it was due to something else. Maybe peer consultation between the dentist and the general practitioner could have convinced me”* (#6).

### Patients’ thoughts about their history of medical and invasive treatments for OFP

All patients tried all kinds of pain medication in the past, including anti- neuropathic pain medication for OFP relief. A lot of these medications did not help, while several patients experienced medications’ side effects (#6.2.1). Patients reported the preference of less invasive therapy before undergoing invasive surgeries. *“Patients with TMD pain should be sent to a TMD team before doing surgery. OFP may very often have to do with the jaw, but this is never considered—at least it wasn’t in my case”* (#5). Patients who underwent surgery found it tough. Patient #9 feels emotional after two Sweet procedures. *“I am actually a little angry that I had the Sweet procedure twice, and my opinion is that a general pain specialist should be wellinformed about all the alternative ways of treating OFP”* (#9).

### Interprofessional team

The simultaneous interprofessional approach was an effective one according to all patients. All patients described getting the same message from different disciplines was effective. *“It would be nice if every doctor looked beyond their own disciplines”* (#7).

## Discussion

The primary aim of this study was to gain more insight in the diagnostic and treatment history of patients with chronic temporomandibular disorder (TMD) pain who had been treated for neuropathic pain. The secondary aim was to get a deeper understanding of the factors that are involved in the possible delay in setting a TMD-pain diagnosis and receiving appropriate treatment. The tertiary aim was to get a deeper understanding of the perspectives and experiences of chronic TMD-pain patients on the possible improvement of various aspects of their diagnostic and treatment journey.

All patients visited numerous health care professionals to seek help for their OFP complaints and received more than one pain diagnosis and several treatments, e.g., medication and/or invasive surgeries for neuropathic pain, which did help only temporarily or partially or did not alleviate their OFP symptoms at all. The data from the interviews showed that OFP patients had different pain characteristics, and the impact of the OFP on their daily life was significant. In all patients that were interviewed, the interprofessional TMD-pain treatment reduced the chronic OFP substantially. This positive result was still present 6 months after the start of therapy. Furthermore, the quality of life for all patients was improved.

One of the most important factors involved in possible delay for setting a TMD-pain diagnosis and receiving appropriate treatment can be the fact that chronic TMD patients sometimes show pain characteristics atypical for TMD pain and more matching neuropathic pain characteristics. TMD pain is usually characterized by a fairly mild intensity and a fluctuating nature. This mild discomfort can escalate to a sharp pain, can include sudden pain increase, and can change rapidly [28]. Orofacial sensory changes in a selective group of TMD patients was reported, like dysesthesia, paraesthesia, anaesthesia, hyperesthesia, and hypoesthesia [15]. A prevalence of around 10% of orofacial sensory changes in TMD patients was found, and it was suggested that muscle compression could be an aetiological factor for these changes [15]. Recently, a new mechanistic pain term was introduced: nociplastic pain, which is ‘pain that arises from altered nociception despite no clear evidence of actual or threatened tissue damage causing the activation of peripheral nociceptors or evidence for disease or lesion of the somatosensory system causing the pain’ [13]. This term seems to fit the altered nociception in some patients with TMD pain, which is still a complicated concept [14–16]. The large number of referrals to different clinicians of these OFP patients could be explained by health care providers not always following all the steps included in the guidelines for chronic OFP assessment and management [29]. These guidelines advise first to examine whether the pain has a dental origin, secondly to examine for a musculoskeletal origin (TMD), and lastly to assess a possible neuropathic origin [4, 25]. In case all steps were followed according to the guidelines, the TMD-pain treatment would have been implemented earlier and in any case before all the invasive treatment modalities including treatments suitable only for neuropathic pain. Another remarkable point is that none of the patients in the present study were aware of bruxism and other oral behaviours before starting the TMD-pain treatment. Possibly, no awareness of a patient of grinding or clenching excludes an examination for a possible TMD diagnosis by health professionals.

According to these OFP patients’ perspective, chronic TMD-pain treatment could be improved by sharing knowledge between different health care providers. Chronic OFP patients often experience overall health care as unsatisfactory and ineffective [30, 31]. Current health care pathways often do not meet the needs of chronic OFP patients despite indications of substantial healthcare usage [22]. The more failed treatments the patient had, the more suspicions arise regarding practitioners and future treatments [31]. This could lead to a possible nocebo effect (i.e., negative expectations of the patient regarding treatment cause the treatment to have less positive effect) when TMD pain patients finally receive TMD-pain treatment [32]. Duration of chronic pain has a negative impact on the prognosis, which underlines the importance of providing effective pain treatment as soon as possible [33, 34]. Some patients in this study had ineffective singular treatments with splints, physiotherapy, and psychotherapies before they received effective simultaneous interprofessional TMD-pain treatment. This finding is in line with other studies reporting that managing chronic OFP patients in a interprofessional setting is the most successful approach [35, 36]. Teaching patients self-management of TMD pain, physiotherapy, splint therapy, and psychotherapy is effective for TMD-pain reduction in case of a interprofessional approach [20, 25, 37–40].

It is known that the BPS model also applies to OFP [7]. Therefore, the International Classification of Orofacial Pain (ICOP) describes the importance of assessing the psychosocial status of patients, and describes the relevance of further research of the BPS model and its clinical relevance to OFP, including TMD pain [3]. According to the chronic TMD-pain patients in the present study, the BPS assessment was not done or insufficiently done by previous practitioners. One of the patients in this study was told several times psychosocial factors were related to his pain, but he did not want to get into that. There can be several reasons a patient does not want to see the role of psychosocial factors in his or her OPD pain. Firstly, it can be confronting for the patient to share private matters about their life with their practitioner. Secondly, it can be difficult for patients to accept that pain has a possible relationship with their own behaviour [41–43]. Thirdly, some patients do not have the cognitive abilities to understand self-management strategies [44]. This rises the demand for more dentists, OFP specialists and pain specialists who are trained and feel comfortable to do a clinical BPS history taking in order to sufficiently educate the patient. In this study, we did not use the classification of the ICOP with regards to primary and secondary TMD. The prior NP treatment and possible diagnosis the patient had in the past are given; that is, if the patient recalled it. All medical information about each patient was gathered from the patients themselves or from the referral letter because we did not have access to other medical systems. The patient and the referrer thought that their own treatment was not helpful enough and thought that there was possibly another diagnosis. After the TMD-pain treatment, most patients believed that the TMD pain was the main cause of their orofacial pain and that the neuropathic treatments were unsuccessful. In this study, the TMD-pain diagnosis can be considered as primary TMD pain with neuropathic pain characteristics, without a diagnosis of neuropathic pain. These ten cases suggest that TMD pain with neuropathic pain characteristics is a possible underdiagnosed cause of chronic unspecified orofacial pain by pain or medical specialists.

### Strengths & limitations

The first strength of this research is that it is the first study that investigates the in-depth experience and perspectives of chronic TMD patients, treated for neuropathic pain, on the diagnostic pathways through the medical system. For this purpose, a narrative approach is most suitable. Another strength is that the state-of-the-art guidelines for narrative research were followed, resulting in saturation of data [27]. The use of modern technology in the COVID-19 period made it possible for this study to be performed online, making the procedure more feasible. One of the limitations of this study was that it is a subjective research because the patients in this research were selected on OFP reduction after interprofessional treatment. Another limitation is the fact that the OFP specialist (JB) had a double role: she treated the patients and was also the interviewer in this narrative research. Since the interviewer has a past caregiver relationship with the patient, the answers given by the patients might have been influenced. On the other hand, some patients possibly felt more comfortable to talk openly about their OFP in in-depth interviews to their own practitioner.

### Implications and recommendations

It is clear that for OFP assessment and management the patient might visit different medical and dental practitioners with varying knowledge and skills in this area [22]. Further research is recommended on the referral pathways and diagnostics of chronic OFP patients, to track down how to improve the implementation of the chronic OFP guidelines. Cross-talk between different disciplines who treat chronic OFP patients can contribute to better OFP diagnostics [45]. Based on the results of this qualitative study, a quantitative study with a larger number of cases could be constructed with the aim of evaluating the usage of the OFP clinical guidelines. A qualitative study could also be constructed, including the narratives of TMD patients with no history of treatment, less visits to medical doctors, earlier diagnoses, and shorter medical histories, which would help in the identification of key points that need to be addressed in the training of professionals.

OFP diagnostics is possibly more complex than simply grouping pain into nociceptive and neuropathic. The third mechanistic descriptor ‘nociplastic pain’ should be considered for the orofacial sensory changes in TMD-pain patients [13]. In cases in which a clear comorbidity can be found between TMD and a nociplastic pain condition, TMD can be considered of nociplastic pain origin. The orofacial sensory changes, aetiological factors, and comorbidities of chronic TMD pain should be further examined to improve diagnosing chronic TMD pain and effective treatment.

## Conclusion

In this study, more insight was obtained in the diagnostic and treatment history of patients with chronic OFP by interviewing 10 patients with chronic OFP. Due to the specific setting of this study, the results might not be generalizable to all TMD-pain patients, but they may be transferable to similar groups of TMD-pain patients with neuropathic pain characteristics. Factors that are involved in a possible delay for setting a TMD-pain diagnosis and receiving appropriate treatment are described. Some chronic TMD-pain patients might show pain characteristics atypical for TMD pain and more matching neuropathic pain characteristics. These pain characteristics could be a reason why health care providers do not always seem to follow all the steps included in the guidelines for chronic OFP assessment and management. Chronic TMD-pain patients experience a long journey until receiving the appropriate diagnosis and treatment. A clinical BPS history taking and a simultaneous interprofessional team treatment were appreciated. Patients concluded that more general knowledge about chronic TMD pain is needed among professionals in the medical and dental sector. This indicates the great need to improve the implementation of the chronic OFP guidelines as well as improve the simultaneous BPS treatment by interprofessional teams.

### Supplementary Information

**Additional file 1.** Topic guide.

**Additional file 2.** Quotes.

**Additional file 3.** Anonymized interview transcripts.

## Data Availability

All anonymized interview transcripts of the data generated or analysed during this study are included in this published article [and its supplementary information files].
