# Supplementary material for: Chronic temporomandibular disorder pain patients with a history of neuropathic pain treatment: a narrative research on their diagnosis and treatment history
Source: BMC Oral Health. 2024 Jan 4;24:22. doi: 10.1186/s12903-023-03796-0 (PMC10768420; doi:10.1186/s12903-023-03796-0)
Supplement: Supplementary file 1 — Additional file 1. Topic guide. [file 12903_2023_3796_MOESM1_ESM.docx]

**Additional file 1 – Topic guide^[[1]](#footnote-1)^**

**Introduction**

Aim: To inform the patient about the interview

- Welcome the patient into the conversation
- Explain the study topic, aims, and objectives of the study
- Clarify the anonymity and confidentiality of the data through the interview, but also during the analysis and reporting
- Explain the recording, duration (1-1,5h), and nature of the discussion, reporting, and data storage
- Explain that the patient can withdraw at any time and that the patient does not have to answer any question (s)he does not want to answer
- Ask if the patient has any questions before we start with the interview and if the patient is happy to continue with the conversation

**Background**

Aim: To obtain contextual information about the patient

- Household composition
- Personal relationships
- Working status: job or no job, what kind of job
- Leisure time
- General health
- Pain in other parts of the body besides the orofacial region

**OFP complaints course before TMD-pain treatment**

Aim: To understand the patient’s pain-related disability and own ideas about the aetiology of the chronic OFP before TMD-pain treatment

- Since when did the patient experience OFP
- What was the location of the OFP when the patient visited the OFP specialist for the first time
- *What was the character of the OFP when the patient visited the OFP specialist for the first time*
- *What was the course of the OFP complaints before the patient visited the OFP specialist for the first time*
- What impact did the OFP have on the daily life of the patient
- What did the patient think what the cause was of the pain before the TMD-pain treatment
- What was patients’ awareness of bruxism and other oral behaviours before the first visit at the OFP specialist

**Medical and dental treatment history before TMD-pain treatment**

Aims: To determine which and how many medical specialists were seen by the patient with the request of pain relief, and to determine what kind of treatments were undertaken aiming at reducing the chronic OFP

- Which medical specialists, in chronological order, has the patient visited for the OFP
- What kind of treatments had the patient for the OFP
- *What were the results of these treatments*
- *What pain diagnosis did the patient get with previous medical specialists*
- What treatment or medical specialist has helped in pain reduction
- Has the patient a suggestion to improve the diagnostics and treatment of his/her OFP

**Interprofessional treatment for TMD pain**

Aims: To find out what the patient thinks about the TMD-pain treatment, and to find out if the treatment helped the patient with pain relief and/or dealing with the chronic OFP

- The OFP specialist made the TMD-pain diagnosis, explained this diagnosis, and planned the treatment. What was it like for the patient to get the TMD-pain diagnosis
- *What did the patient learn from the OFP specialist*
- What did the patient think of the splint
- What did the patient learn from the orofacial physiotherapist and/or speech therapist
- What did the patient think of the OFP specialists’ advice to go to the psychologist
- What did the patient learn from the psychologist
- *What did the patient think of the interprofessional treatment (three or four practitioners in the same period)*
- What moment was the patient aware of bruxism or other oral activities during the TMD-pain treatment
- What does the patient think is the cause of the OFP after TMD-pain treatment
- Give a percentage that represents the pain reduction at the end of TMD-pain treatment
- Which part of the TMD-pain treatment helped the most in reducing the OFP complaints

**Current OFP condition**

*Aims: To find out how the current OFP condition is, how the patient thinks about possible pain relapse, and to end the interview on a positive note*

- How is the current OFP condition
  - Are there still moments when the OFP is really bad and needs painkillers/medication, exercises and/or splint
- How does the patient explain relapse of the OFP
- *Was the patient informed that there could be relapse of the OFP*
- *What does the patient still use from what (s)he learned with the OFP specialist and does the patient still use her/his splint*
  - *e.g., preventive wearing the splint*
- *What does the patient still use from what (s)he learned with the orofacial physiotherapist and/or speech therapist*
  - *e.g. practice the exercises of the orofacial physiotherapist*
- *What does the patient still use from what (s)he learned with the psychologist*
  - *e.g. reducing stress to prevent getting relapse of the OFP*
- *What does the patient think after the TMD-pain treatment of the OFP specialist’s advice to go to the psychologist*
- Does the patient like to say something to medical specialists about diagnosing and treating OFP
- Does the patient have any other points to bring up after the completion of the interview

**In conclusion**

*Aims: To repeat confidentiality, and to ask again permission to the patient if the interview can be transcribed and used for research purposes*

- Thank the patient for her/his time
- Explain confidentiality again
- (S)he can call us if (s)he has any further questions
- Ask permission to transcript the interview (anonymized) and if the interview can be used for research

1. Italicized text represents additions to the topic guide during the interviews. [↑](#footnote-ref-1)
