# Supplementary material for: Chronic temporomandibular disorder pain patients with a history of neuropathic pain treatment: a narrative research on their diagnosis and treatment history
Source: BMC Oral Health. 2024 Jan 4;24:22. doi: 10.1186/s12903-023-03796-0 (PMC10768420; doi:10.1186/s12903-023-03796-0)
Supplement: Supplementary file 2 — Additional file 2. Quotes. [file 12903_2023_3796_MOESM2_ESM.docx]

**Additional file 2 – quotes**

#1.1.1*“Imagine you’ve got two live wires - if you hold them together you get sparks, but once they’re away from each other it stops”* (#4).

#1.1.2 Patient #1 described his OFP as a blocked nose. *“It developed very slowly, stinging behind my eyes, cheek and nostrils. There were days I could no longer feel my ear, it was like feeling a piece of cardboard on my ear”* (#6).

#1.2.1*“Talking is a big part of my job and as the day goes on you suffer more [from the OFP], which results in losing the ability to talk. Besides talking, I also suffered while eating, drinking, and laughing”* (#4).

#1.2.2*“It caused a chronic pain that no one could identify. People around me were shocked when I told them how long my chronic pain lasted. *patient cries*”* (#3).

#1.2.3*“When you tell your child, you will try living with OFP as long as possible, but you know living with it [OFP] is not something you can keep up indefinitely, and you consider suicide”* (#8).

#1.2.4*“I suffer from OFP mainly during the night. You have a bad night’s sleep which affects your whole psyche, because if you are tired you can handle it [OFP] even less. I ended up in a vicious circle that got worse and worse”* (#6).

#1.2.5*“Eventually you cannot handle it [daily life] anymore. You don’t sleep, eat, drink, work or do and sports anymore. Even taking care of your own child is almost impossible. You always need help from others, which is tough”* (#7).

#1.3.1 Patient #6 thought his OFP was referred pain from the neck. *“So for me it was very clear that arthrosis in the neck was where my OFP was originating, but afterwards it turned out the origin was something else”* (#6).

#2.0.1*“I had quite a lot of visits to doctors and other hassles; you do not want to know how long that list is”* (#8).

#2.1.1*“According to the dentist and implantologist, everything looked good, but the pain remained. After that, I was referred to the neurologist by my general practitioner. He diagnosed trigeminal neuralgia”* (#4).

#2.1.2 *“Once I said to the neurologist: can you send me to someone who can teach me how to relax my jaw? But I was never told that this was a possibility”* (#5).

#2.1.3*“You could have brought a whole truckload of paracetamol, but it would not have made any difference”* (#2).

#2.1.4*“I also had tramadol and oxycodone once and tried all kinds of morphine-like tablets to keep the pain away. When the OFP was really bad, you were so depressed”* (#7).

#2.1.5*“The problem with amitriptyline was that my blood pressure went down. So when I was on my bike I almost fell off because my blood pressure was so low. Then I thought this just isn’t working any longer, I have to do something else”* (#6).

#2.1.6*“It [Janetta operation]* *was a very invasive operation. It took a lot of time, and since the operation I can no longer perform regular work, I cannot handle that anymore”* (#2).

# 2.1.7*“Eventually I was referred to the OFP specialist at my sister’s request. The neurosurgeon wanted to do more surgery, and then I said: ‘I don’t want that, because I don’t trust it.’ I had an awful feeling about it [the proposed surgery]”* (#7).

#2.1.8*“After three weeks, I was discharged from the hospital. When I got home, I was in pain again. I called the neurosurgeon and told him this. He said the operation had succeeded, and he told me to go to the dentist to check if any of my teeth were the problem”* (#9).

#3.1.1*“The pain was finally given a name; it was a relief after 17 years of pain. I assumed the person I was talking to [the OFP specialist] knew what kind of treatment was necessary”* (#7).

#3.1.2*“I thought it [psychosocial anamnesis] was quite hard, because the OFP specialist discovered quite a lot of personal things about me, such as family circumstances, stress factors, etc. I had to go through all that”* (#9).

#3.2.1*“I talked with the psychologist about my OFP and about my whole life. Slowly, I learned to look different to myself and I started to cope with certain things differently. I received the [occlusal] splint from the OFP specialist. The overloading both physically [bruxism] and figuratively [psychological distress] was already getting less. The physiotherapist taught me how to relieve the tension in my jaws. They [members of interprofessional team] all worked perfectly together, despite their different professional fields”* (#7).

. #3.3.1*“It was the first time that someone really listened to what I had to say, and that someone asked me questions. This was very important for me and this was when in my opinion the whole story came together”* (#1).

#3.4.1*“I noticed that the splint allows me to relax my jaws more. At first I had to get used to it, because the splint gave me an annoying feeling. But now I find it annoying when I am not wearing it”* (#2).

#3.4.2*“I miss the splint sometimes, when I do not wear it. Then I go back to the bathroom to get it. It is like a seatbelt; when you are not wearing it, it feels like something is missing”* (#6).

#3.4.3*“If you had only given me the splint, that would not have been enough for me”* (#7).

#3.4.4*“The [occlusal] splint has made me more aware of what I am doing with my teeth”* (#10).

#3.5.1*“Your body has to re-learn how to relax. I did not even know how to position my jaw in a relaxed state. Physiotherapy really helped me discover that”* (#8).

#3.6.1*“I have become very conscious of the position of my tongue. The way I used my tongue during swallowing was wrong”* (#3).

#3.7.1*“In my perception, psychologists were spiritual people, and I do not like that much”* (#2).

#3.7.2*“I found it hard that the TMD pain treatment was not only about receiving a splint and physiotherapy, and hoping the pain decreases. No, I also had to go to a psychologist”* (#6).

# 3.7.3*“At first, I thought I won’t do that. I know for sure I am not going to tell everything, but when I was at the psychologist’s office, it actually went really well. I felt comfortable about telling everything and it was really informative. I never thought psychological distress could have such big influence on your whole body”* (#9).

#3.7.4*“OFP is not only a physical complaint - the tension comes from somewhere. There were a lot of consultations with the psychologist, which were really intense, but I thought it was very helpful”* (#3). Patient 7 felt really comfortable about visiting the psychologist. *“I did not find it confronting at all. I actually laughed with that man and we had really nice and open conversations”* (#7).

#3.7.5*“I learned to build in peace for myself. I was the type of person who never stopped, always thought everything and everyone around me comes first, and that I never needed to rest”* (#2).

#3.7.6*“Due to the high sensitivity, I feel too much on a high level. I did not know I felt this, I just saved it and blocked it”* (#6).

#3.7.7*“I tend to second-guess what someone else thinks about me. Then I am bothered what someone else might think of me. I learned this is something I experience, and I am the one who has to do something about this”* (#4).

#3.7.8*“When I was 17, I came out as lesbian. That was the first time. Then I went back into the closet, because apparently I was not yet totally ready to handle it yet. I am just more a brooder”* (#7).

#3.7.9*“I learned from the psychologist that I have to try to be more open; I have to dare to say things. At first, I was really against this [being more open] and I felt nervous about it. However, when I opened up, it felt really nice. *patient cries*”* (#9).

#3.7.10*“I recognized it: sexual abuse, but I had always denied it. I took a peek at it, but I never acknowledged it happened. When I finally acknowledged it, I was in shock for days”* (#10).

#3.7.11*“I learned a whole lot from the psychologist. And since then, some of my mood swings and anxieties are gone, and I am better able to be myself”* (#10).

#4.2.1*“I still remember I visited the OFP specialist for the first time. She told me: ‘You do not even want to know what you are doing with your mouth.’ I had no clue”* (#3).

#4.2.2*“Suddenly, you start to realize what you are doing all the time, and then the battle starts - to stop doing that. Stopping chewing gum was hard for me as well”* (#8).

#4.2.3 *“I had to think about my facial posture. I had to learn how to handle my movements better. For example, I did not have to wet my lips all the time. I found that very difficult”* (#9).

#5.2.1*“I learned from the physiotherapist how to massage my own jaws. I have to say, however, that I don’t do that anymore. I think I’llgo back again [to the physiotherapist] and pick it up again now we have had this conversation”* (#3).

#6.1.1*“When you look at the symptoms and pains, practitioners are apparently unable to say ‘it is grinding your teeth’”* (#10).

#6.2.1*“When the OFP was really, really bad, I needed a lot of medication. I could actually no longer function with this high dose of medication”* (#5).
