# Supplementary material for: Chronic temporomandibular disorder pain patients with a history of neuropathic pain treatment: a narrative research on their diagnosis and treatment history
Source: BMC Oral Health. 2024 Jan 4;24:22. doi: 10.1186/s12903-023-03796-0 (PMC10768420; doi:10.1186/s12903-023-03796-0)
Supplement: Supplementary file 3 — Additional file 3. Anonymized interview transcripts. [file 12903_2023_3796_MOESM3_ESM.docx]

Raw English interview data

Background of participants

**Participant 1:** XX years old, husband, cohabiting, children living away from home
Work: retired, formerly human resource manager
Activities: walking, cycling, gardening, choir singing, volunteering
General health apart from facial pain: good, but under the supervision of a cardiologist. Heart surgery in 2016. Pain due to worn out back

**Participant 2:** XX years old, wife, married, children 10, 8 and 6 years old
Work: 21 hours/week as carer for the elderly, previously more irregular work as carer for the disabled

General health apart from facial pain: Rheumatoid arthritis since 2006, frequent abdominal complaints since 2014. Takes medication against gastrointestinal ischemia. Had several TIAs, suspected scleroderma. Spasms in oesophagus for a long time. She uses biological medication for rheumatism and scleroderma

**Participant 3:** 49 years old, wife, married, two adolescents and two children living away from home
Work: teacher, two days a week. Previously business, Business Studies diploma
Activities: gardening, cycling, reading and walking

General health apart from facial pain: Good, restless legs and tires quickly since contracting TB at the age of 28

**Participant 4:** XX years old, wife, married, two daughters living away from home
Work: complaint mediator in a large hospital, three days/week
Activities: running, family history research

General health apart from facial pain: good. Recently melanoma and lymph nodes removed with favourable results

**Participant 5:** XX years old, XX, married, two children
Work: two days/week volunteering in a nursing home and two days/week babysitter for grandchildren
Activities: swimming, supporter of footballing son, reading, walking, cycling

General health apart from facial pain: High blood pressure with medication. Once had kidney stones, some wear to joints in hands and feet

**Participant 6:** 59 years old, male, married, 2 children living away from home
Work: started with the military police and has been with the police since 1985, the last 15 years as an analyst in forensic investigation.
Activities: (long-distance) walking, gym, visiting a physiotherapist, photography

General health apart from facial pain: Fit, healthy body weight. However, trigger points throughout the body that sometimes lead to pain but are under control with targeted exercises

**Participant 7:** XX years old, wife, married, son of six.
Work: Bookkeeper, studied International business and languages
Activities: walking with the two family dogs

General health apart from facial pain: Good. In 2019, pneumonia with severe signs of fatigue and high levels of inflammation

**Participant 8:** XX years old, wife, single, two children living away from home

Work: freelancer in marketing and communication field
Activities: tennis, Argentine tango, swimming, formerly a lot of running

General health apart from facial pain: good. Has suffered from chronically inflamed Achilles tendons for a long time. Finally treated with massage and injection of blood. Occasional headaches

**Participant 9:** XX years old, wife, married, children living away from home
Work: retired
Activities: games, puzzles, reading, salsa dancing

General health apart from facial pain: good. At the moment, most likely a heel spur. Occasional knee pain, according to participant this is osteoarthritis

**Participant 10:** XX years old, male, divorced, 2 children - partly - resident, 1 living away from home

Employment: consultant for consulting in the field of employment
Activities: gardening, music, motorcycling, sailing, electronics

General health apart from facial pain: good. Occasionally uses light antidepressants and sleep medication. Had a lot of headaches. Occasional bowel problems. Frequent earaches. Sore throat radiates to back, tinnitus, dyslexic

Facial pain complaints and -impact before TMD treatment

‘The trick is to fall asleep because once you're awake, the pain falls back on top of you like a blanket...’ Distressing stories about how facial pains disrupts/disrupted the lives of ten Dutch people.

'For ten, fifteen years I had a constantly blocked nose, especially at night', begins
**Participant 1.** 'That's why I woke up all the time. When I sat up for a moment, it opened up but after I lay down again the complaints repeated themselves. It also seemed as if I had continuous inflammations in the mouth and pain in the jaws. Occasionally, it moved through to the right ear, then that whole side was sensitive. So, I slept badly but still had to get up early, as I was responsible for a large group of people at my work. That miserable situation had repercussions on work, concentration, colleagues and my own household.'[1]

Suicide disease

**Participant 2** even calls it a suicide disease. 'Not that I've had those thoughts, but I can imagine that if you had to deal with it for years. I ended up in a better place pretty quickly. However, it did take a year.'**[2]**

**Participant 2** started getting a lot of pain in 2016. 'All at once, out of nowhere. Chewing was especially painful and cold wind.'**[2]**

Waking up to the pain; getting really tired of that all-pervading pain in your face... **Participant 2**: 'Headaches often go away after an aspirin or a paracetamol. You could turn up here with a whole truck of paracetamol, but it wouldn't make a difference. The pain is just constantly present. Especially left in the jaw, especially near my nose.'**[2]**

Lonely

'I have to think a lot about people who have corona and feel very alone', says **Participant 3.** 'I was 28 years old when I got TBC, I experienced loneliness very deeply. The facial pain caused such a chronic pain that, in my case, I couldn't bring anyone home. I did express it to others, but you feel alone because no one really recognises it.'**[3]**

**Participant 3** fortunately never got woken up from the pain. 'Every day, especially around four o'clock, it started and continued until I went to bed. I felt it especially in my lower jaw, teeth and gums, but also in the upper jaw. It felt like a chronic headache.**[3]**

Electricity

‘Imagine, you've got two live wires. If you hold them together, it starts sparking. Once they're away from each other, it settles down.’ This is how **Participant 4** describes her pain. ‘It's mainly on the right side, in line with the upper jaw.’**[4]**

There are also periods when she doesn't experience pain. 'Even during very stressful times. And then, for some inexplicable reason, it's there all at once. One day more, the next less. It can last for a week or two or three and then it disappears again. And so it comes and goes. The only thing that became clear was that the cold and a cutting wind played a role.'**[4]**

**Participant 4** does not wake up from pain and also experiences few problems in the morning. 'It does interfere with my daily routine. As a complaints mediator, talking is a big part of my job. As the day progresses, it gets more difficult and then I can't do anymore. Apart from talking, I have trouble eating, drinking and laughing, so I spontaneously open my mouth.’ **[4]**

Dark and quiet

**Participant 5** had been in pain for 10 years before she came to the gnathologist. 'In the beginning it was really bad; then I didn't want to go anywhere, because I was afraid I would get it while there. After a few years I got over it; I didn't want the pain to control my whole life. But it's always on my mind. When it's really bad, there's not much I can do. Then I'd get despondent: when will this ever stop? Once I told my husband that I didn't want to carry on any longer, but that's when you don't know what to do anymore. I feel that he and the children understand. The first years, I fortunately didn't have it at night, but later on I did and it was severe , especially in the cheek between the nose and ear. When everything is dark and quiet... Then it's even worse! It's such an annoying pain, you can't explain it.’ **[5]**

Piece of cardboard

**Participant 6** got pain especially at night. 'It's like an electric shock, which burns for a very long time afterwards. It's on the right side, on the underside of the jaw towards the nose. Very slowly it creeps in everywhere. Stabbing behind the eyes, in the cheek and the nostril. I've had days, that I didn't feel my ear at all, as if there was a piece of cardboard on my head. These were the strangest phenomena, actually. Just because it's in the face...'**[6]**

**Participant 6** had the facial pain usually within a period of about three months. With a peak that then subsided, also because of medication. Pain attacks sometimes lasted a few minutes but could just as well last for an hour and a half. I was lying with my finger in my mouth at night, because there was a certain nerve spot there; as long as I pressed it, the pain disappeared.'**[6]**

If the complaints continue leading to a bad night's sleep, this will affect your entire psyche, **Participant 6** continues. "I ended up in a vicious circle that got worse and worse. And I was afraid. My mother had had a facial pain, she’d had an adhesion of the nerves in her face. Those were really terrible stabbing pains. After a Jannetta surgery, it went well for a while but then it came back. My continual nagging, also stabbing pain was not there, but in the back of my mind I was afraid that it would end up that way.'**[6]**

Social isolation

Already at the age of 17, **Participant 7** developed facial pain. From my point of view, the pain was mainly on the lower left side of the jaw. It would go in a straight line downwards and then all the way to the eye. It started with small stabbing pains as if someone was pinching me. At first, three times in one day, then ten times. The frequency became higher and higher, the duration longer and longer, and at one point it seemed as if I was being electrocuted.'**[7]**

Then you go back five steps, so to speak, in your actions, **Participant 7** states. 'You don't want it to get worse. But you can't stop it. Once it starts, you're actually already too late and your whole social life is turned upside down again. At some point, you just can't do it anymore. You don't sleep anymore; you don't eat anymore; you drink almost nothing; you haven't worked for three or four months. You don't exercise anymore; taking care of your own child is almost impossible; you always need help from others and that's just very hard. A social isolation. Sleeping was best. Until I turned over in my sleep and the attacks came back...’ **[7]**

Nettle

During the first two years it wasn't so bad, but after that I didn't know what to do anymore," says **Participant 8**. At first it seemed like I had a very salty liquorice in my mouth that I had to swallow or suck on. I got the idea that if I chewed on something, it wouldn't bother me for a while. At one point I walked around 24/7 with gum in my mouth... I also had headaches and sore cheeks but especially my tongue. It reacts to all kinds of food: salt is not good; acid is not good; this is not good; that's not good... I constantly had the idea that I had a kind of nettle in my mouth. Or hydrochloric acid. It was so dominant that I had no room for other things.’ **[8]**

The impact on her life was huge. 'I had told people at work that I was suffering a lot and they accepted that I was always chewing gum, but it's still weird for me that you're sitting in a meeting like some kind of chewing cow.'**[8]**

In the end she couldn't concentrate either. 'It takes an enormous amount of energy. I didn't feel like doing anything either. At one point I said to the children: I'm not going to walk around with this for seven years. Because when I looked on the internet, I saw people who had been suffering this for that long. I ate Spanish chillies to get through the feeling of pain. My gum chewing turned out not to be the best thing either, but okay. I was like: I can't keep this up. And if you tell your child that you'll try this as long as possible but that it's not something you can keep up indefinitely ... That's awful. You don't want to hear that from your mother.'**[8]**

Also for **Participant 8**, sleeping was the only solution to avoid the pain for a while. ‘The trick is to fall asleep because once you're awake, the pain falls back on top of you like a blanket. You might as well wake up and think for a moment that there is nothing wrong, take a sip of water and then suddenly it can be all wrong again. I would have done anything to get rid of it.'**[8]**

Not herself

In **Participant 9**, the pain started at least 20 years ago with two molars in the left lower jaw. I went to my dentist before that but he couldn't find anything in the x-ray that explained the pain. Then he pulled those two molars. My symptoms remained until it seemed as if I was being tortured. A burning sensation, but also painful electric one.'**[9]**

Sometimes things went well for a number of years and then she got pains again. It's strongest in the morning. Is that because I'm clenching my teeth in my sleep? It could be.'**[9]**

Also for **Participant 9**, the impact was enormous. 'I could do quite a lot of things, but there was no spontaneity anymore. I don't want to say that I have become unbearable, but I'm also not a ray of sunshine in the house anymore. I couldn't be myself, that's it.'**[9]**

Bomb

Finally, **Participant 10**. From the age of fifteen he experienced pain and even that seemed to be just toothache in the beginning. The tooth I was grinding on hurt. And I had stiff jaws. I never made the connection between my teeth and the facial pain including the numbness on both sides of my face around the ears. It seemed like a slight ear infection, sore throat or inflamed lymph. The doctor and dentist couldn't make that connection to the teeth either. I had to find out for myself through injury and embarrassment.'**[10]**

“Bubbling ears like there was an inflammation in them"; magnified loud incoming sounds... **Participant 10**: 'If someone touched a spoon against a cup, it could sound like a bomb was going off.
"I expressed it to others, but you feel alone, because no one recognises it. At times, therefore, I just walked away from noises, which is sometimes a disaster for those around me. I sleep only briefly. At night, I can wake up from tinnitus, sounds in my ears. An ENT doctor told me that grinding my teeth was very much related to that.'**[10]**

**Participant 10** often experiences less pain in the morning but it builds up during the day. Usually his pain returns three to five times a week. 'I have various kinds of pain. One is dull, the other sharp. Ear ache sometimes comes on strongly; a sore throat is worse when I talk. One pain is debilitating, no paracetamol helps against it. The doctor doesn't allow me to take Ibuprofen anymore because of the effect on my gut. Every now and then, in the afternoon between three and four, I just finish the day because it hurts so much. There is no suitable medication or I am not receiving it. My children notice that daddy is in a lot of pain, so I have to try and spare them too. My productivity decreases, my energy management decreases, my mental well-being decreases, the tendency to withdraw increases. People in a lot of pain have little need for people anyway, I think.’ [10]

Streamers:

*'I did express it to others, but you feel alone because no one really recognises it'*

*When everything is dark and quiet... Then it's even worse!*

*I ended up in a vicious circle that got worse and worse'*

*Once it starts, you're actually already too late and your whole social life is turned upside down'*

*'At one point, I walked around 24/7 with gum in my mouth'*

*‘And if you tell your child that you'll try this as long as possible but that it's not something you can keep up indefinitely ...'*

*'When the x-ray didn't explain the pain, he pulled those two molars. The pain remained...’*

*'You could turn up here with a whole truck of paracetamol, but it wouldn't make a difference'*

Cause of facial pain according to the participant before visiting a gnathologist

Those desperate searches! Some participants thought their first complaints were simple toothaches. What followed was an escalation of complaints with an accumulation of referrals. As **Participant 10** explains: 'I've pretty much covered the entire medical circuit to get rid of those pains.’

**Participant 10** briefly visited a natural healer. He spent a number of years on bioenergetics to make himself feel better. Visited a psychologist, a psychiatrist, an ENT doctor several times... The facial pain, the teeth grinding, the biting, not being in contact with your emotions, not being able to express yourself, swallowing your emotions, not moving enough, not relaxing enough ... The whole song and dance has been heard dozens of times!' **[10]** He's never been referred to a neurologist.

'The dentist just removed a small part of the tooth on which I was grinding so that there was no more pressure on it' **Participant 10** continues. The pains remained and grew worse. Sometimes they were gone for a while, and then they came back with full force. After an X-number of months another fractured surface occurred, which started to hurt. In the end, pieces of a bridge and crowns also started to break off; teeth broke off or just burst in half. The dentist gave me splints in several shapes, sizes and types. I became quite addicted to those things. The dentist also referred me to a gnathologist."**[10]**

Food allergy?

Because her tongue was reacting strongly to food, **Participant 8** thought she might have a food allergy. I didn't know any more so I went through a whole list of people who all looked at me a bit blankly.'**[8]**

Her saliva was tested. 'It turned out to be pathologically too acidic and... ...I don't remember. I was advised to put soothing walnut oil in my mouth; otherwise there was no real treatment. I went to a throat, nose and ear doctor, who thought it might be a nerve disease that could continue to develop. It wasn't. In the end, I was given some kind of ointment, for a mouth fungus, which I reacted to very badly. I was in tears when I put that in my mouth. So I stopped doing that. I was with a Chinese acupuncturist and an organic eating centre. They didn't really come up with anything out of the ordinary.'**[8]**

The dentist had no idea either. **Participant 8**: He did say that he had had a man in his practice who, after eating an ice cream or a pastry, had lost his taste for five years until it suddenly returned'**[8]**

Even an oral surgeon couldn't reduce the pain. 'He was part of a club investigating these kind of things. But they couldn't resolve it either. At a certain point the GP called and said that he had followed a training course with a gnathologist. "That story made me think of you. Do you mind if I refer you? It's all the way to Nijmegen, though!” If it was on the other side of the world, I would have gone!'**[8]**

Bleach

Because she eats fruit at four o'clock and around that time the pain usually starts,
**Participant 3** thought there might be a connection with food, for example fruit, acids or coffee. Nothing came out of that. Then she thought about... ...Bleach. 'As a child, I fell over a lot. This caused my front teeth to discolour. That bothered me a lot. Four years ago I bleached them for the second time. I thought a sensitivity in my mouth had arisen because some of the fluid might have leaked. My doctor wasn't able to deal with my symptoms. I had those three years not knowing what to do. I read a lot, looked for what it could be, a real journey of discovery. It took a long time before someone could help me with it. Finally, a year and a half ago my new dentist referred me to the gnathologist. I first had to go to the periodontist to rule everything else out.'**[3]**

Hadn't thought of that

**Participant 1** reported to a doctor several times; the ENT department of the Radboud UMC made scans. 'I was always told that nothing was wrong. Even the dentist didn't know what to do with it. I did wear a bite plate for a year and a half because she thought I was grinding my teeth too much. I hadn't thought about that at all. Very soon my jaw started to do other things.'**[1]**

Participant 1 ended up at the gnathologist after a referral by a new GP in the summer of 2018.

Merry-go-round of medicines

**Participant 2** was initially referred to the dentist. 'The dentist filled a small hole but it was the doctor who started dealing with the facial pain. I then tried Lyrica - pregabalin - and amitriptyline for a while. Because of my scleroderma story there was also contact with the rheumatologist and then a neurologist. He triggered a facial pain protocol. They made an MRI scan which showed that the nerve was very close to a blood vessel. The neurosurgeon who operated on me in 2017 also saw this. Although of course you first have to recover from that heavy operation, after that it seemed to be heading in the right direction. Until during a dentist's checkup I sat with my mouth open for too long and the pain came back. Then I went back to the dentist and family doctor and I also had contact with the neurosurgeon again. He suggested that I should go to a gnathologist. There was quite some time in between and in the meantime I was given five other medicines: Carbamazepine I believe, also Vento.... Each time something different, three quarters of a year in total. The pain stayed. I just thought it was pure bad luck.'**[2]**

Periodontist

**Participant 4**: 'I always had a lot of problems with my jaw and was often visiting a periodontist. First, bone was transplanted, then I got pillars, a bridge and finally implants. From then, about ten, eleven years ago, I got the first signs of pain. According to the dentist and implantologist, everything looked good, but the pain remained.’

She suspected that nerves had become damaged or hypersensitive during the treatment. ‘I went to the neurologist through my GP. He diagnosed trigeminal neuralgia, which means facial pain. He also said that there was actually no treatment except medication to suppress the pain. I was given oxcarbamazepine and played around with a minimum - 600 - and a maximum dose of 22. I discussed what the surgical possibilities were with a neurosurgeon. With the help of an MRI, he stated that there was no conclusive indication for a Jannetta procedure. The blood vessel that pressed against a knot was the draining vessel and not the supplying one, which would give a conclusive indication. Or it was the other way around, I don't remember.'**[4]**

Grandma

**Participant 5** first thought it was toothache. 'I had pain on the left side, exactly at the position of the molar on which a crown had been placed X-rays showed nothing. Yet it hurt me every time and kept nagging at me, even after I received antibiotics because they thought that maybe my cavities were inflamed. I asked a neurologist to send me to someone who could teach me to relax my jaw. I got no reaction to that. When it really shot into my face, I knew it was facial pain. I recognised it from my grandma who used to suffer from it. The GP had to laugh a bit about my certainty but immediately sent me to the neurologist; after that I ended up with the neurosurgeon. Nothing came out of an MRI. We tried different medications. The Trileptal oxcarbazepine works best for me.'**[5]**

**Participant 5** wanted more. 'At a pain clinic, I had an extensive conversation about the Sweet. I didn't have it because I didn't like it that much myself. But I knew what was possible in case there was really nothing else.'**[5]**

There then followed a Jannetta operation. It didn't help at all. The following year I was in a lot of pain and talked to another neurosurgeon about a motor-cortex stimulation. Because I only have complaints for a short period of time during the year, he didn't want to do it. "It's still very much in its infancy; I'm not recommending it now.” Then he asked me if anyone had looked at my jaw. No, they hadn't. Through him, I ended up at the gnathologist.'**[5]**

Cause versus effect

The search... Eight years ago, **Participant 6** visited the GP because of neck problems. 'Very slowly, more things started to creep in. In the end, you don't know what's the beginning and what's the end; what is the cause and what is the effect.'**[6]**

According to the GP it was only some stresses, **Participant 6** continues. 'Be glad you don't have anything; you just have to learn to live with it", that's how it was told to me. Without being referred or even having an x-ray. She referred me to her primary psychologist. I went there twice but we didn't make any progress together. A physiotherapist treated the neck but said after ten sessions: "I give up, I can't make any more progress.” You just end up in such a void!"**[6]**

But he kept going anyway. An orofacial physiotherapist [*was a manual therapist*] observed that the first and second vertebra had a blockage. After three attempts he also stopped because he couldn't get any further. He wanted to refer me to a physiotherapist with psychology or something. After the experience with the GP's psychologist, I had become averse to it. So I went home again: and now? I work all day at the computer and that just didn't work anymore, my neck was completely blocked and painful. I went looking again.'**[6]**

Trigger points

**Participant 6** ended up with a chiropractor who also did dry needling. 'He found that my body had a lot of trigger points. They can cause all kinds of things in the neck and face. Photographs showed that cervical vertebrae were indeed worn out and trapped nerves were a cause of pain in the back of my head. I also noticed it: when my shoulder was pressed on, it moved up through my face.'**[6]**

So it wasn't just tension, concluded **participant 6**. 'Twenty years ago I had a burn-out, those symptoms were now totally out of the question. It was only during the TMD treatment, with the psychologist, that I revealed that I am highly sensitive. If you don't know you're feeling too much and you're just storing it, it gets blocked. So tensions certainly played a role for me, but I didn't realise that until later.'**[6]**

The chiropractor cleared the mess in the neck pretty quickly. **Participant 6**: 'In the beginning, by old-fashioned cracking. A dentist asked if I shouldn't go to the gnathologist but at that moment I thought that the pain in my neck was caused by osteoarthritis.'**[6]**

Water bag

The ailments increased. **Participant 6**: 'In my face, the pain was the worst where a crown had been put in. On the left-hand side a crown had been placed on the molar at exactly the same level. That's where the facial problems started. But there was nothing to be seen in the x-rays even though it hurt me every time and kept nagging me. By the way, and that's where I end up with TMD, it turned out that there was a fracture on the left side of that tooth and that it was completely broken on the right side. Apparently it was in the TMD that I bit them to pieces.'**[6]**

The nerves on vertebrae 3, 4 and 5 on the right side were blocked against the neck pain. **Participant 6**: 'A test blockage went very well but during the treatment for permanent blockages my head and back of my head were completely on fire. I didn't feel anything after the injection, but once at home everything remained heavily numb. It seemed as if the back of my head was a big water bag. And that is, to some extent, still the same. So they treated something that shouldn't have been treated. They took x-rays that showed the needles were well placed and that this could never affect my occipital nerve, but with me it was the case. So apparently, I'm put together differently than the average person. I have suffered a lot because of this and still do. The pain on the right side went away for nine months, but then it came back again. I do suffer less from cold wind on the right side now, but the side effects are less pleasant.'**[6]**

**Participant 6** also suffered a lot from a burning sensation in the face and neck. 'Because this was actually a nerve pain, I was referred to the neurologist. He didn't want to operate, so prescribed amitriptyline instead. My blood pressure went so low that I almost collapsed next to my bike. It just didn't work anymore, only at that moment I didn't know what to do. A colleague with similar neck complaints hadn't suffered from it for years thanks to a pain clinic. Then, at my request, I was referred to it. And that opened my eyes a lot.'**[6]**

Introvert

**Participant 7**: At the age of 17 and for six years after, until I found out what I had, I went to the dentist during a period of pain. I even had a molar pulled while the dentist said it wasn't the problem. Indeed, the pain didn't diminish. Once a very deep cavity was being filled when something must have been touched, the tears were running down my cheeks. My jaws were hit while playing sitting football when I was about twelve years old. At the Centre for Special Dental Care they checked my salivary glands. They also checked my teeth but found nothing. Everybody always said it was stress but I didn't believe that at first, I just did my thing.'**[7]**

In the meantime, **participant 7** calls herself an "extreme introvert, a thinker". 'In that period I was trying to come out of the closet about liking women. Apparently, at one point the last straw came .'**[7]**

She was given Carbamazepine 200 mg and gabapentin 300 mg. 'For years I went on with it. I came off it when the pain was gone and started taking it again, as soon as the misery returned. Just as I did with my new GP, with whom I have been with for ten years now. I got tramadol, oxycodone and all kinds of morphine-like tablets when I was at a real low ebb because of the pain. At one point he sent me to the neurologist. After all the brain tests he sent me to the neurosurgeon who did a Jannetta surgery in 2016. I thought: "Yes, now I'm sorted!"**[7]**

The following year the complaints came back. **Participant 7**: My sister, who was studying ayurvedic medicine at the time, said it might have been my jaws. Then the neurosurgeon sent me to the gnathologist. At my sister's request. Otherwise he would have liked to do another operation but I didn't have a good feeling about it.'**[7]**

So there's something other than a Jannetta operation, **Participant 7** now knows. 'I had never heard of TMD, though. It's nicer to go into something like that, than something invasive like an operation. Because it's just much harder to recover from a process like that. After the operation I had enormous headaches for weeks, even a little cough would hurt my head a lot. They placed these sponges; sometimes I'd think: maybe that's not right at all'**[7]**

Allergic

In 2011, the GP of **Participant 9** discovered that she had trigeminal neuralgia. 'Pain on the third branch of the left side. I got Ibuprofen 600 and amitriptyline. That didn't work well in the end so he referred me to the neurologist who prescribed Carbamazepine. We then went on holiday and I got a terrible rash all over my body. A have no idea how many medications the local GP gave me but, all in all, it didn't help. Once at home, I also got it all over my face. The neurologist was shocked: "You're allergic to Carbamazepine!” That same day, she referred me to a dermatologist. He prescribed all kinds of ointments and medication, but it didn't go well. Then they sent me to a pain specialist.'**[9]**

An anaesthesiologist suggested the Sweet procedure. **Participant 9**: 'I got the first one in early 2012. After that, it went well for at least three years until I got a lot of trouble again. My second Sweet procedure only worked for three months. Then my husband and I read about the Jannetta surgery on the internet.'**[9]**

She got it in 2016. 'During the operation, the neurosurgeon discovered that I had not one but two entanglements in my brain. All in all, I had to stay in hospital for three weeks because I lost cerebrospinal fluid twice. Once I was home, I had the pains again. The neurosurgeon told me the operation was successful and referred me to my dentist, maybe it was a nerve from a root canal? He didn't find anything and sent me to a prosthodontist and restorative dentist. He measured me for a top splint. That didn't help. I also got eleven dry needling treatments. That did help at first, but later it didn't.'**[9]**

Eventually, she ended up with a new GP. ‘He sent me to the VU where I was prescribed Lacosamide as Vimpat. Up to now, that has been the best remedy for my facial pain. I started with two 50 mg pills and built that up to 5 times 50. The VU anaesthesiologist referred me to a gnathologist."**[9]**

Streamers

*'The dentist just removed a small part of the tooth on which I was grinding; the pain remained and just got worse’*

*'If it was on the other side of the world, I would have gone!'*

*'I had those three years of symptoms without knowing what to do’*

*'I just thought it was pure bad luck'*

*'According to the dentist and implantologist, everything looked good, but the pain remained’*

*‘The Jannetta operation didn't help at all.’*

*'It seemed as if the back of my head was a big water bag. And that is, to some extent, still the same'*

*'Everybody always said it was stress but I didn't believe that at first, I just did my thing'*

*'The neurologist was shocked: "You're allergic to Carbamazepine!”'*

The experience of a TMD pain diagnosis

And then you hear you're suffering from TMD. "TM what?” But then. 'Is it really that simple? I thought. Then you're gonna have to deal with that.'**[2]** 'I was surprised, I had never really thought about it, It felt like an enlightenment because finally there was something that made me understand my symptoms.'**[3]**

The participants are quite unanimous in what this turnaround meant to them. ‘I was glad there might be a solution for which I had been whining for all those years.’**[1]**

‘It got the feeling that this could be the right direction. I still think so.'**[4]**

'At last I heard what I had always been thinking. I felt, most of all, relief.'**[5]**

'It had been said to me a couple of times by a dentist, so it wasn't really a surprise.'**[6]**

'The pain finally got a name after 18 years. Then you know which process to use and that was a great relief.'**[7]**

'What helped enormously is that at least you know there's a way. There's also a bit of recognition about it. It's just super frustrating that you're doing it to yourself because it's really awful.'**[8]**

‘At first I went to the dentist for teeth grinding and in the meantime I really understood that my problems had something to do with it. TMD is just a different terminology. The biggest eye-opener was that the gnathologist made a connection between teeth grinding and sexual abuse. I thought I’d got through it and then bang, bang, bang, it came back like a gunshot.'**[10]**

Switching

It meant that the participants had to switch from a somatic to a biopsychosocial approach. 'It was a bit challenging, but I also heard a lot of things that I recognised in myself. Of course, it's part of it.'**[5]**

'I found that hard. Not just a bite plate and muscles... No, you should see that psychologist. And I didn't see the TMD team as the ones who could solve my problem.'**[6]**

'In the beginning I had to really think about it, because I seriously felt it wasn't because I was stressed or tense. Now I know: by looking at things differently, you get a completely different sense of things. The teeth alone wouldn't have been enough for me. The three-pronged approach has helped a lot.'**[7]**

'I found that quite tough because they could find out a lot of personal things, including family circumstances. I had to go through it whether I liked it or not.'**[9]** She continues: "I never thought that stress could affect your whole body so much, including your face, including your jaws. Yes, stress is an element you have to deal with differently."**[9]**

Streamers:

*‘Finally, there was something that made me understand my symptoms’*

*'At last, I heard what I had always been thinking'*

*'The pain finally got a name after 18 years. Then you know what process you have to take.’*

*'It's just super frustrating that you're doing it to yourself because it's really awful'*

*'The biggest eye-opener was that the gnathologist made a connection between teeth grinding and sexual abuse'*

Experience of treatment by a multidisciplinary team

Suddenly you run from the gnathologist via the physiotherapist and the speech therapist to the psychologist... During the TMD process, each participant received a tailor-made package of counsellors. This multidisciplinary team was visited once, or usually several times, within one period of an average of more than three months. How did that work out?

'I'm very satisfied with this process, **Participant 9** starts. The gnathologist did all kinds of tests and evaluations; the physiotherapist provided exercises and the psychologist was simply a relief. I never expected to say that. I also think the team was all on the same page. Apart from the gnathologist who told me to use a splint during the day and the physiotherapist said at night. Anyway, that doesn't mean they weren't on the same page.'**[9]**
**Participant 1** received treatments from a psychologist and an orofacial physiotherapist apart from the gnathologist. 'This also made the situation clearer for me, for example because everyone indicated the importance of the mouthguard. It was the first time I was listened to attentively and asked questions. Not "we'll make a scan and it will come back", and then be standing outside again within ten minutes. Everybody knew my background in advance and immediately took the matter in hand. Information was exchanged continuously. 'We'll share it in the workgroup, so we know exactly what's happening, what progress there is and where there are still gaps.” It was just beautiful. Slowly but surely we were getting to the point we wanted, brilliant!'**[1]**'It's nice that everybody's working together like this. Then you immediately see where the pain points are, which you can do something about. Sooner than when you do everything one after the other.'**[2]
Participant 3:** 'If I had a question about something, it was often fed back to the gnathologist. I think it's very important that practitioners know each other so as not to be judgemental towards the other. I experienced the team as a unit and that was very pleasant.'**[3]**

Link

'The gnathologist explained very well at the time about working with a multidisciplinary team, says **Participant 7**. 'I'm open and pretty easy so I just got stuck in. I just assumed that the knowledge present was sufficient to start and end the process properly. A neurologist or neurosurgeon also does a good job, but that's one thing and that's what you have to do. In this case, three people are acting in their own field at the same time, but they are working together towards one point, I like that. The gnathologist gave me a some help with the mouthguard. It came to light that I have psychologically literally started to bite things. You only realise that when you talk about it together.’

The psychologist took over this part. **Participant 7**: 'Slowly you learn to look at yourself differently and to deal with things differently. So literally and figuratively the biting becomes less and less. In addition, the oral therapist teaches you the tricks of her trade to reduce tensions in the jaw. As a result, you simply have a lovely process in which you work together perfectly. It has helped me a lot!'**[7]**

'The whole package was clear to me and I think the right people were picked out for me.'**[6]**

More holistic

**Participant 4**: ‘Three tracks... These three disciplines must reinforce each other in order to get a good picture of what I can do, what the effects are and what the long-term benefits will be for me. The multidisciplinary approach is therefore a good move to try to find the connection. To avoid the stress, to build up less tension and to work with the jaw at the same time. The awareness at several points, that tension can still be a trigger for these pain attacks.'**[4]**

**Participant 8**: ‘Everyone told me about their piece of the same cake. I had more trouble with the psychologist because it didn't add anything for me. After that I went to talk to someone else.'**[8]**

‘I think it's the right thing to do. Everyone works on one theme from his or her own discipline, and the related themes that emerged were also included. A more holistic approach than just: "you've got a toothache."'**[10]**

Intensive
It was a very intensive period but the participants liked it. It is precisely the fact that you're very busy for a short period of time that makes you very consciously fight your facial pain and also do well at home. As I had to go to Nijmegen for the treatments, I could very often plan different things in one day. That was very busy but easy to do'**[5]**
I had to travel two and a half hours every two weeks to get there and the same to get home again. Once I got home, I was completely exhausted. On the other hand, it was a fairly short period of time, only about three months.'**[2]**
'It really demands everything from you: three, four months of hard work. That whole combination did make me very aware of the whole process. That it's not just the tongue pressing, but also why I do it and in what situations. I found it all connected together very logically.'**[3]**
**Participant 6:** "I enjoyed having it all at once. You also want to move on, so if you first have to finish one process and then go to the next... It shouldn't take too long, because you just want to finish something. Otherwise in two years' time you'll still be deliberating about all sorts of things. No, it all works neatly and smoothly after each other, I could get anywhere very quickly.'**[6]**
'I was very happy with it. Because it just didn't work out, I had stopped working so I was really like: the more, the better; if it doesn't work out now, I'll go crazy, it's as simple as that. It's very intensive in terms of time, but it's really good to do together and it was really necessary.'**[8]**

Streamers:

*'It was the first time I was listened to attentively and asked questions'*

*“We'll share it in the workgroup, so we know exactly what's happening, what progress there is and where there are still gaps”*

*'I think it's very important that practitioners know each other so as not to be judgemental towards the other. I experienced the team as a unit and that was very pleasant.'*

*‘I think the right people were picked out for me’*

*'The multidisciplinary approach is therefore a good move to try to find the connection'*

*'A neurologist or neurosurgeon also does a good job, but that's one thing and that's what you have to do’*

*'A more holistic approach than just: "you've got a toothache"'*

*‘It is precisely the fact that you're very busy for a short period of time that makes you very consciously fight your facial pain and also do well at home'*

*'It really demands everything from you: three, four months of hard work’*

Experience with the gnathologist

'I had never heard of a gnathologist...'**[7]** How many Dutch people have? Still, this specialist is central to the multidisciplinary team around TMD complaints. **Participant 1**: ‘It was the first time I had been listened to attentively. She told me where things went wrong, what could go wrong, all that kind of stuff. "Do you even know what you're doing to your jaw?” We've started working with all these tools .'**[1]**

**Participant 3** remembers that during her first visit, the gnathologist told what she did with her mouth. 'I was totally unaware of it. No one had ever told me how weirdly I was doing things. I had seen for years that my tongue had ridges. Only I never realised it was because of tongue pressing.’

On holidays or weekends, **Participant 3** doesn't suffer much from it. 'As soon as I go to work, I start feeling a pattern developing in my mouth. I ran my tongue mainly along the bottom and, a little less, along the top of my teeth. Nowadays it's really focused on the bottom. Even when I'm gardening, I feel my tongue pressing against it.'**[3]**

When **Participant 3** was being treated by the gnathologist, she hyperfocused on sucking her tongue. 'I had sucking spots in the back of my mouth, they were like blisters. My GP had it checked with the ENT doctor. He confirmed the story of the gnathologist.'**[3]**

**Participant 5**: "The gnathologist taught me to take better care of myself. That's really not always easy for me, but I've changed.'**[5]**

'As a first step, she taught me to accept that I'm a biter. I needed her persistence. That it was indeed so and that it still is.'**[6]**

'I try to watch what I do with my mouth during the day now. That's not always easy, because it's learned behaviour you keep doing.'**[7]**

Three minutes

**Participant 8** had to bite her molar for three minutes for the gnathologist. 'I got one command after another! She told me how many times I was doing that, all day long. You become a lot more aware of what you're doing. Stopping with the chewing gum was also hard, because it caused me to go crazy. But yes, I also understand why I had to do it and that my body had to learn to relax again. She showed that there was a way .'**[8]**

I learned that I have to think about my facial posture," says **Participant 9**. 'That I don't have to wet my lips all the time and other things. I learned a lot from that, because that also prevents me from clenching. The annoying thing is, I have such a problem with a dry mouth that I keep putting my lips back together and wetting them.'**[9]**

**Participant 10**: 'She has recently fitted a fantastic new mouth brace. I'm definitely also thinking about the insight of making some connections between toothache and possible sexual abuse. It is not scientifically based, it is not substantiated, it is also dangerous, because it is not empirical, scientifically proven. I don't usually get people explaining that there is a relationship between tooth grinding and possible sexual abuse. But she's an expert on the subject. I liked those conversations. I also learned to keep a constant eye on those teeth. When someone's lying in bed next to me: “do you hear me grinding my teeth?”’ **[10]**

Streamers:

*'No one had ever told me how weirdly I was doing with my mouth'*

*'As a first step, she taught me to accept that I'm a biter. I needed her persistence'*

*‘At one point she let me bite my molar for three minutes. I got one command after another'*

*'I don't usually get people explaining that there is a relationship between tooth grinding and possible sexual abuse. But she's an expert on the subject'*

Experiences with the bite plate

One participant gets a dry mouth, another discontinued use because of pain. In general, the participants range from satisfied to lyrical about the bite plate or the mouthguard or the splint. 'That mouthguard is my support and crutch! I once slept without it but my jaw was in bits early in the morning, I never used to notice that.'**[7]**

And that, while his first experience with a mouthguard was not exactly positive. 'Before I did anything, I bought one of those mouthguards from a shop. Dip it in hot water, bite down and you get the right shape. Only, every morning I kept finding it somewhere else in the bed. I thought: if I have to bite into something, it's certainly not that. I was pretty stubborn about it.'**[7]**

**Participant 1**: In the beginning, I wore the mouthguard for a very long time. My jaw soon started doing other things. If I get problems now, or I feel it coming on, then I pop the mouthguard back in. All I get is a terribly dry mouth. Nevertheless: along with the physiotherapist and the jaw physiotherapist, it was the most important thing that helped with pain reduction.'**[1]**

Rest moment

**Participant 2** was unaware of the tooth grinding. ‘That's what I got the mouthguard for. I still use it almost every night. I notice that it also gives my jaws a bit more rest. In the beginning I had to get used to it, but if I don't have it now, I find it annoying.'**[2]**

**Participant 3** is also satisfied. 'I notice that with the mouthguard the pain increases less quickly. It provides a moment of rest for your mouth. I do notice that I really do gnaw on it at night. In the beginning, I would put it in during the day, but I don't feel like that anymore. When I'm gardening, I think about it because when I'm raking up the mess I notice: there goes my mouth again. I now wear the mouthguard on average once every five days, only at night. I don't sleep so well with it, so I don't tend to do that to myself every night.'**[3]**

It really doesn't bother me, but I also don't know what it's doing," says **Participant 6**. 'I use it all the time unless I really have a cold, then there's so much mucus that it doesn't sit comfortably in the mouth. Like a seatbelt, I miss it when I don't have it in.’**[6]**

Very satisfied

**Participants 5, 7, 8, 9** and **10** talk very enthusiastically about the splint. 'I always have it in at night and carry it around in the car. I feel great relief because then I can't clench my jaw... That just helps.'**[5]**

**Participant 7:** 'That mouthguard is my support and crutch. I got it in 2017. In the beginning, you talk very strangely because the old position of the jaws is not at all correct, you only notice that when you put the mouthguard in. Then I wore it for a long time every day, every night, and during work, all the time. At a certain point I weaned myself off it, because it felt better and better. To relieve certain tensions in my jaw, I still wear it every night.'**[7]**

'That mouthguard helped enormously,' says **Participant 8**. 'I still have it in. I put a lot of force on my jaw and the mouthguard helps to calm it down.'**[8]**

**Participant 9**: 'I started with the top splint. Then I don't press so much. I think it’s because I then feel more pain when I start clenching. I am also working on the lower jaw so now I have two splints. I use them every day.'**[9]**

‘With the mouthguard in, I have a much greater awareness of what I'm doing with my teeth. During the day, without the mouthguard, I notice that my teeth or my jaw are in a slightly different position. More open with less tooth contact. I am addicted to my plate; I don't want to wake up in the morning with screaming pain from grinding my teeth. If there is a certain sensation, I notice that I'm building up tension in my teeth, then the tooth grinding is definitely going to start. Every now and then I put in that brace during the day. For example during car and motorcycle riding.'**[10]**

No success

**Participant 4** gave up. 'I'd been wearing the mouthguard for a long time, but not for the last few months. In a period with a lot of pain, I can hardly touch my mouth. After a whole night, the mouthguard seems to have sucked itself in and then it hurts a lot to get it out.'**[4]**

Streamers

*'My jaw soon started doing other things'*

*'In the beginning I had to get used to it, but if I don't have it now, I find it annoying’*

*'It provides a moment of rest for your mouth'*

*'In the beginning, you talk very strangely because the old position of the jaws is not at all correct, you only notice that when you put the mouthguard in’*

*‘ I have two splints and I use them every day’*

*'I am addicted to my plate; I don't want to wake up in the morning with screaming pain from grinding my teeth'*

*'After a whole night, the mouthguard seems to have sucked itself in and then it hurts a lot to get it out'*

Experience with the orofacial physiotherapist

'When I first saw the jaw physiotherapist, she already knew my background. She took the right approach, I got a good explanation and information sheets with what I had to do'**[1].** 'At times I say "banana" in combination with a blowing exercise for 20 minutes in a row. Motorists must think: "what's that crazy man doing?!” It has to do with the jaw joints. And you have to them massage, from the inside.'**[9]** Experiences with the orofacial physiotherapist.

Relaxed jaws, pain reduction. A luxury for people who've been experiencing the opposite for years. **Participant 2**: 'After my gnathologist pointed out how much tension I put on my jaws, I started paying attention and indeed, it was true. When I came back from the jaw physio, I sometimes noticed that they were a little more relaxed and less painful, just fine. The exercises I learned there help even more.'**[2]**

'The jaw physio showed with very simple exercises where the problem was and what you can do with it', says **participant 6.** 'With that one simple treatment she did, I thought: shit, now I feel it. I knew, it's all these things that come together and this was one of the causes. By tackling it, the problems in my face have been pretty much solved. Massaging the jaw edges, the face itself, behind the ear, down... Those are the exercises I do when those toothaches come back.'**[6]**

**Participant 4** concluded to have - got - the most from the physiotherapy within the TMD process. 'Feeling good in the jaws where you might notice those swellings, those nodes as I call them. Massaging the skin above your ears, your jaw in your neck... I firmly believe that tension in that area triggers a pain attack. But certainly what my mouth was doing. As long as I notice anything that is tense again, I immediately ask the physiotherapist to watch and feel with my exercises.'**[4]**

Oh, yeah...

**Participant 5**: ‘Oh dear, I'm doing it again... But now I notice it! Yes, I really learned from the physiotherapist how to relax my jaw. Letting it hang, creating space. Every day, I look for the trigger points in the underside of my jaw.'**[5]**

In the beginning, **Participant 7** and her physiotherapist went into her oral behaviour a lot. 'In the swing that I had when opening and closing the jaw, there was a kind of S shape. We tried to get rid of it. We also talked about relaxing in plenty of time. For example, by massaging the jaw in the shower or bath. Because we talked about it together, I suddenly realised: oh yeah!'**[7]**

The oral therapist said in the first interview that she had hardly ever seen a person so open and positive in the treatment, **Participant 7** glows. 'Very nice to hear, but from me: finally I was helped! I think, if you yourself are more negative about the treatment because you've had facial pain for so long, then you just don't get the realisation that quickly. And you don't get rid of your symptoms.'**[7]**

'The physiotherapy with dry needling and massage has in any case loosened everything up again. It's still not completely gone, but it has calmed things down a bit. And it helped to recognise my body again, that there is another way. I learned to relax muscles myself, loosen them. On the Visapure from Philips there is a massage attachment, that will also help sometimes. And showering!'**[8]**

No success

Physiotherapy was not a success for **Participant 10**. 'I learned a very intensive technique that I couldn't get ingrained in my daily pattern. It gave her a moment of relief, if she did it for sure, but the effectiveness was short. 'I bought a book about how it all works exactly, but also that ebbed away. I no longer use anything I learned at the physiotherapist. Later I went to another physiotherapist six times to massage away muscle tension from the jaws, but that didn't work either.'**[10]**

Discipline

Do the other participants keep practising, even if the symptoms have subsided? **Participant 3**: 'In the beginning, I very faithfully massaged my jaws and did exercises. In my diary where I keep track of how the pain develops, I see that I haven't massaged myself for a year now. I'm doing so much less. Now that I think about it, I'll go to the physio again.'**[3]**

'In February my head was pretty full again and I got the pain back very lightly. Together with the therapist I went through exactly what I have to do. Within a month the symptoms were gone again.'**[7]**

Streamers

*‘At times, I have to repeat "banana" for 20 minutes in combination with a blowing exercise’*

*'The jaw physio showed with very simple exercises where the problem was and what you can do with it'*

*'If I notice anything that is getting tense again, I immediately ask the physiotherapist to take a look at me'*

*‘Oh dear, I'm doing it again... But now I notice it!'*

*'I learned a very intensive technique that I couldn't get ingrained in my daily pattern'*

Experience with the speech therapist

Some participants were sent to the speech therapist during their TMD process. It had an effect.

**Participant 3**: 'It's natural for everyone, but apparently not for me to put the tongue against the palate. I pressed mine hard against my lower jaw, especially when swallowing. I still do that at times, especially when I'm working. It was mainly with the speech therapist that I became very aware of my tongue position, a revelation! Yes, with the speech therapist, I learned most about what I do or did and that it should be different.**[3]**

**Participant 5** was also always having problems with his tongue. 'With the speech therapist, I learned with exercises to eventually relax my tongue. I had to learn where to put it or leave it.'**[5]**

**Participant 8** also learned a lot from the speech therapist. What really helped me was the speech therapist, the manual therapist with the dry needling, and the osteopath. I still go to the latter once every 14 days.'**[8]**

Streamers

*'It was mainly with the speech therapist that I became very aware of my tongue position, a revelation!'*

When the participant was told to consult a psychologist

Exaggerated: with a toothache to the psychologist? That last remark indeed surprised several participants. The general mood was also that they went for the full package. 'You go for the treatment, you hear the process, then you commit 100% to it.'**[4]**

Generally, feelings were mixed. 'I had a really hard time with it, I wasn't crazy or anything!'**[1]**
'I was like, oh boy, what are we going to do?'**[5]**
**Participant 4** was not frightened: 'I think it's good for everyone to talk to a psychologist at some point.’
'A bit of nonsense," said **Participant 2**. 'In my experience, psychologists were a bit vague and I didn't have much time for that. It was the wrong image.’ She got upset when she realised that the gnathologist was right: she was grinding very badly! 'Only then did I realise that there was only tension on my jaw and that I also had to do something with my tongue. Then I should try the whole package, I thought.'**[2]**

Still good
The hesitant participants sounded positive afterwards. **Participant 6:** 'I just decided to listen to what they had to say and then see. I think it was a very good combination and that the right person was chosen for me. I thought she was a great person. She specialises in HSB, high sensitivity. She finally confirmed that I'm highly sensitive. So we suited each other well for that reason. It went very well and we only needed three conversations to be able to close.’
**Participant 9**: 'At first I thought: bye, bye, I'm not doing that. I was sure that she wouldn't be able to get me to reveal anything. At a certain point I did it anyway, and I have to say that it actually went well. I dared to say what I had to say anyway.'**[9]**
'My psychotherapist gave me tools to gradually learn tips and tricks to use in my life. So that you can deal with how you are. At times, I found it difficult to change gear when my work wasn't going well. Then he said: "maybe you can deal with it that way, too?"**[7]**
**Participant 10**: I've seen a lot of counsellors in my life. Part of my profession consists of a grey area of assistance. I know all the tricks, layers, states and methods which that can cause a great deal of irritation because I know where it's going. Well, not with this psychotherapist. He's one of the better ones. I don't want to say that I always went there gladly because there were very painful moments, also a lot of extremely painful moments. But I have a great deal of respect for him.’

Streamers

*'You go for the treatment, you hear the process, then you commit 100% to it'*

*'I had a really hard time with it, I wasn't crazy or anything!’*

*'He gave me tools to learn tips and tricks to carry through my life. So that you can deal with how you are'*

Experience with the psychologist

The psychologist pushed me very slowly and surely in the direction where I had to go, what I had to think about and that I had to think about myself...'**[1]** What do the participants think about their psychologist afterwards?

**Participant 8** is negative about the few times she visited the psychologist. 'Everyone, of course, tells their piece of the same cake. With the psychologist, I had more trouble with that because it didn't add anything for me. I'm not someone who is constantly looking back.'**[8]**
If she had felt it would have helped, she would have persevered, **Participant 8** continues. 'In this story, the big difference between the osteopath and the psychologist is that with the former I don't have to go digging into whether my childhood was happy or not. That's all very well, though, of course, everyone's got their own issues. It is more about how I react to things. As the osteopath says: "stress normally peaks and troughs, but with you, it just builds up.” We're playing with that now and I'll get much further with that than to theorise about how it all came to be. I wouldn't do it any other way now, because that's the choice you make. That's the way you are as a type. It's very important to teach your body to reduce stress.'**[8]**

Super

'Particularly the process of thinking about myself and being more open about it was very important to me', says **Participant 1**. He also learned to ignore some things. 'I thought it was great how it went with her, that helped me the most in reducing my pain. She asked and asked and slowly but surely she got to the point where she wanted to go. That made me feel really good.'**[1]**

**Participant 2**: ‘I'm one of those people who always keeps going. And always thinking that the other thing comes first. That I never need my rest. I've written down a sentence from the psychologist and often think about it: "Do you have to do this now?” There are several ways to read it: you have to, do you have to do this now, do you have to do this, and do you have to do this now?'**[2]**

"I found it quite a lot, and very intense. But, of course, that tension has to come from somewhere. It became clear that there was still some old pain. It's good to relate that to each other. She also told me: accept that this piece belongs to you and will continue to belong to you. That helps make it okay that it's there from time to time.'**[3]**

At the helm

'That psychologist was fine', finds **Participant 4**. She was glad that various points of attention came up. 'It gives me peace of mind to let things slide past me. Does it bother me? Am I going to do something about it? So that you're more at the helm to regulate your own feelings. I don't know if it'll make me feel less pain or not. How you feel in life, what you've been through and how you deal with things, certainly has something to do with stress, tension and the jaw joint. Very confronting! During my work I have been telling others for a long time that thinking about how someone else thinks about you creates stress. Make it negotiable or check it. Now I noticed: I am practically ignoring my own advice!'**[4]**

**Participant 5** only visited a psychologist once. 'It was a pleasant conversation but we both came to the conclusion that I just have to keep doing my thing. I've had it for so long and I'm working on it well, I don't lock myself up at home anymore. I do my best to let go even though I don't always succeed.'**[5]**

High sensitivity

In **Participant 6**, the psychologist diagnosed high sensitivity. 'Physically, of course, that creates tension. If you don't understand that all those feelings that come over you do something to you, it stays in your body. On top of that, I'm a policeman. "Don't be silly, carry on..." so I blocked it out myself. Looking back, that old burn-out came out of this.'**[6]**

During that period he had almost two years of therapy. 'I learned a lot of skills at the time, only to forget a bit about them. Now, I just had to pick them up again, especially being aware of what you're doing and why you're doing it. That's why we were finished after only three sessions. What was always part of it was: don't leave things in limbo, deal with them. Then it's solved and you don't have to think about it anymore. Also to challenge certain thoughts: is it really like that? Then you think: okay, I'm overreacting again. With some relaxation exercises, it's all going fine again. Don't leave it in limbo but take it on. I'm actually doing pretty well.'**[6]**

Tools

**Participant 7**: "You don't realise things until you talk to someone about them. Otherwise, you won't get rid of those complaints. I had to learn to handle myself in order not to fill my bucket too high and especially not to let it overflow. I was always there for everyone, but that was a one-way street, so I stopped that. Nowadays, I try to say right away what I think, feel and think. I also learned to let go a little earlier. Difficult, because I like to finish things, I set my standards very high. So I have to take a step back sooner. I'm still in that process. Not worrying so much about things. Having a different way of starting a conversation when I find something awkward. That's what the psychologist gave me. "Fine, if that's what you think, that's it.” Done...'**[7]**

'At first I was afraid: they're going to dig into my life again,' explains **Participant 9.** 'But it was actually good. Because you don't really have anyone else to whom you can say what you've been through and what you may have always kept to yourself. I have to try to be more open, to dare to say things. I was very much against that, but, it sounds crazy, now I really like it. She also showed me two websites with relaxation exercises. In the beginning they hurt but I liked that. Also read what pain can do to you.'**[9]**

Tension

**Participant 10**: 'At first we had to wrestle. Do I know him, or does he know me? But when I look at the effect: some of my mood swings and fears are gone and I'm better able to stay by myself. I had to deal with a very big burnout and a divorce. And there was a confrontation with my parents. That was a tremendous amount of tension that built up in three, four, five months. The fear of just accepting that I would cut off 100% of my contact with my parents, that they would not accept what I was going to say. And that half or the whole family would know, with many of them trivialising it, I would lose them. I still feel the condemnation, that I confronted those poor old people with that. I've been through a lot in many ways: wanted, unwanted, undesired... One of my children recently made a suicide attempt; then I noticed that the stress in my jaw area was increasing again. Shit happens all the time, but I think I'm getting through it in a good way. I may not be aware of all the stress. I wouldn't know in God's name how to break through it any more.'**[10]**

Streamers:

*'She asked and asked and slowly but surely she got to the point where she wanted to'*

*‘The big difference between the osteopath and the psychologist is that with the former I don't have to go digging into whether my childhood was happy or not'*

*‘Accept that this piece belongs to you and will continue to belong to you. That helps make it okay that it's there from time to time'*

*'How you feel in life, what you've been through and how you deal with things, certainly has something to do with stress, tension and the jaw joint'*

*'It was a pleasant conversation but we both came to the conclusion that I just have to keep doing my thing'*

*‘Challenge certain thoughts: is it really like that? Then you think: okay, I'm overreacting'*

*‘You don't realise things until you talk to someone about them'*

*'At first we had to wrestle. Do I know him, or does he know me?*

Conscious of mouth habits now

'You can feel what you're doing, but changing it, that's where the crux lies. So I can feel what I am doing, but always too late', declares **Participant 8**. Just about every participant used to be unaware of tooth grinding and jaw clenching, but now they are. But quitting is quiet another story.

**Participant 8**: 'In itself, it's not rocket science. Not biting your jaws or not sucking your tongue, how hard can it be? Very difficult! I still can't get my tongue to relax. It's still at the bottom; if I raise it, it's going to irritate me right away. And when I am concentrating and stressed, I unconsciously start clenching my jaws again, it's a bad habit. But then things explode immediately. So it's straight away value for money, I'd say. But too late, always too late.'**[8]**

**Participant 3** is also fighting with her tongue position. 'Now my tongue is at the top for 80 percent of the time, but I can't always favour the opposite. Not because of an exaggerated stress, but because of being busy and not aware of what I am doing in my mouth. When I'm in front of the class, I'm completely absorbed by the children. I want to be there for every child and at that moment I'm completely unaware of what I'm doing in my mouth. That causes me to push my tongue down again. Also during a meeting, I can feel the pressure increase. But if my mouth is bothering me after a day, now I know what's causing it. That gives me some peace; I accept that it's there and I know that it will go away again. I used to be afraid: what if I have this for the rest of my life, I still have a long way to go. The total package has made me very aware of the complete process and that there is a lot behind it. And that you can get ahead of some situations. For example, I used to worry in advance about a fully planned work week. I am now a little less stressed about upcoming situations beforehand, the psychologist helped me with this. And I plan my day well so that I have enough rest.'**[3]**

Increased awareness

'In any case, I now have a much greater awareness of what I do with my teeth,' **Participant 10** now knows. 'During the day, I notice that even without a mouthguard, my jaw is in a slightly different position; it is more open and I have much less tooth contact.'**[10]**

Unfortunately, his pain hasn't diminished. 'It's not a question of not believing that I can't make a causal connection. Otherwise, it would be gone by now. What I can explain, however, is that what has built up over the years is not simply one, two, three and gone, but perhaps slowly ebbing away. I lead a pretty healthy life. I meditate, I work out, I have a nice relationship and, of course, every now and then there’s a lot of stress. Yes, I think it's an expression of the tension. Some people get abdominal pain, others get lower back pain or migraines or whatever. My whole process was based on tension, especially considering the sexual abuse. I had the silent hope that, by dealing with that, the pain would disappear. Well, it hasn't. There's definitely a relationship between tooth grinding, ear, throat, lymph nodes, headaches, radiation pain. I have no way of influencing that in any way.'**[10]**

Being alert

Another similarity is that the moment the participants feel something, they are also in pain. **Participant 4**: 'That's where it starts. I know that I have to do something right away. Calm down, be alert and get help.'**[4]**

She has become convinced that tension in the entire complicated jaw joint plays a crucial role. 'That that tension is caused by incorrect movements when chewing, talking and sleeping. I have to find some solace in this. More than that, there is a blood vessel pressing against such a nerve node in my head', says **Participant 4**. 'I think I still have that tension. A lot is still unclear in this area. Where does this pain come from? The relationship between tension and symptoms is not 1 to 1.'**[4]**

Setback moments

'I used to notice that I was very tense and always tired in my jaw but the real awareness came through the TMD team', starts **Participant 5**. 'I still do my jaw exercises every day. And when I push my lower jaw forward again, I often think: oh no, do it differently. I still benefit a lot from that. I will have to keep doing that too. During the holidays I get very tense in the car. I've heard that to get over that, I have to put a bag over my head.'**[5]**

**Participant 5** thinks he is still active at night with the front teeth. 'I don't notice that during the day, but they are completely worn out!'**[5]**

TMD pain generally has relapse moments. **Participant 5** didn't know that. 'Last November, I had it again for a while, but certainly not as bad as before. Of course I don't know if it will stay that way. If I have it, I'm now more at ease. Just keep doing my exercises and things like that. At the moment, it shoots into my lower jaw for a moment, but in a minute it's gone again. For me this is unique, because when it used to shoot into my lower jaw, it would last fifteen minutes.'**[5]**

Horrible video

'It's that my husband recorded a horrible video on which I could see what my mouth habit was like. I never really knew that. I know that when I have a dry mouth, my tongue runs along my teeth', observes **Participant 9**. Because of the bad habits, I've probably put a lot of demands on my jaw joints.'**[9]**

She still has her mouth habit. 'I have such a problem with that dry mouth, that I just put my lips back together again, wet them and so on. And when I'm watching something exciting or want to win a game, I have to be careful not to make those backward movements again. I really have to be conscious of that. With my tongue towards my palate. Try it, and you won't be able to do anything crazy.'**[9]**

**Participant 2:** 'What's wrong now, do I have a lot more tension? I pay good attention to it. Once, I wanted to visit a jaw physio in my neighbourhood, but they were on holiday. After that period the symptoms were fewer, so I left it like that.'**[2]**

Mix

**Participant 6**: 'I think it's been a constant mix of causes. That from the mutual tensions between one comes the other. When I have tension in the face, it really is TMD with clenching as one of the causes. If it's on this side, it radiates into my sleep and that's a different story. And this one, which runs here and is the most sensitive... That's not so much TMD, that's more tension in the neck and the expression of it.'**[6]**

The moment his face bothers him, he knows where to feel and where to act. 'So, I keep an eye on it: what am I doing with my mouth and face, am I clenching? That awareness is there. But I can't prevent it. I'm biting my teeth to pieces. The pulling from the mouth and what I then do... It's so cunning, it's very hard to get out of it. Of course, it's not like you're looking for what the hell you've got, while everyone's saying something else. Yeah, you don't get much further than that.'**[6]**

**Participant 7**: 'I no longer fear the facial pains like I used to. I live with the thought that it never quite goes away, but we can make it bearable. It is limited, bearable and just liveable. With a pause, I can now continue to work, eat, talk, drink and do my social things.'**[7]**

Streamers:

*'Not biting your jaws or not sucking your tongue, how hard can it be? Very difficult!'*

*‘That gives me some peace; I accept that it's there and I know that it will go away again'*

*'Calm down, be alert and get help'*

*'It's that my husband recorded a horrible video on which I could see what my mouth habit was like'*

With a pause, I can now continue to work, eat, talk, drink and do my social'

Tipping point in facial pain reduction

In the end, what mainly reduced facial pain? **Participant 1**: 'The bite plate caused my jaw to move in the right direction. In addition, physiotherapy and jaw physiotherapy. And mentally, it was the psychologist. All three were useful, definitely.'**[1]**

**Participant 2**: 'At first, it was the operation, but then the pain returned. With the TMD team, came the awareness.'**[2]**
**Participant 3** mentions the mouthguard and the referrals to the psychologist, speech therapist and orofacial therapist. 'They really suited me. Super important, because otherwise it wouldn't have worked.'**[3]**
'I think I got the most out of the physio,' says **Participant 5**. 'The awareness about what I do with my jaw also helped, and the splint.'**[5]**
For **Participant 7** the turning point came with the gnathologist. 'The nice thing is the three-pronged process. First, the mouthguard for literally and figuratively biting away the symptoms. Then talking about it with someone and at the same time getting the massage treatments so you can relax your jaw.'**[7]**
**Participant 9** mentions the medication as a turning point. 'And what the gnathologist told me about how my mouth should behave. Followed by the exercises from the physio and the conversations with the psychologist.'**[9]**

Percentage of facial pain reduction and anti-neuropathic medication after TMD pain treatment

The big question is to what extent have the pain symptoms decreased? Are the participants still taking medication?

According to **Participant 1**, his facial pain decreased by at least half immediately after the multidisciplinary treatment of TMD pain. 'Slowly but surely, it's continually decreasing. Occasionally, when there is tension in the house, it goes up and down. Then the mouthguard goes back in, I do my exercises and things like that. After two or three days the symptoms go away. This morning, I happened to be thinking about it. I was lying on my side, my whole nose was nicely open and I could therefore breathe easily... done! Nowadays, I sleep about seven hours a night. I do wake up once, but in nine out of ten cases I fall back to sleep fairly quickly.'**[1]**

In most cases, the pain has been reduced by as much as 60 to 70%, claims **Participant 2**. 'In case of stress, sometimes at the very beginning, I think: shit there it is again. The pain is not as extreme as it used to be. Back then it went on for hours, days in a row, now it's a relatively short period of time, half a minute or so. I don't take pain medication anymore either.**[2]**

Less anxiety

**Participant 3**: 'When I left the gnathologist, the pain was 100% gone. In daily life, I can't keep that up. In the holidays it's about 30% present. And during a busy working day, it can shoot back to 100%. But I'm less concerned about it. Chronic headaches are still there, but much less. I'm not afraid of it anymore, I now know what it is and that gives much less stress and anxiety.'**[3]**

"I reckon half the pain's gone," says **Participant 4**. 'If necessary, I'll take medication, which will have a fairly quick effect . Only a small amount though, because it makes me terribly ill. I start with 300 mg, which is of course nothing because I need 600 mg if I don't want to be in pain. The first 14 days, I'm really sick, vomiting in the morning. But then it subsides a bit. Once, I was able to increase it to 900 mg, but that was all I could manage. It's better to have a few seizures than to be so sick all day.'**[4]**

Completely satisfied

'I didn't have it too bad when I joined the TMD team. When I left, I wasn't completely symptom-free either, but the pain was at least halved. I don't think I'll ever get rid of it completely, but if I can do it this way, I'm actually completely satisfied.'**[5]**

**Participant 5** is now using three and a half Trileptal per day. 'I tried to reduce it to two but then I got symptoms again in November. I agreed with the neurologist to keep in touch about medication every year.'**[5]**

'It is very difficult to indicate by what percentage my pain has disappeared,' says **Participant 6**. 'I still take medication - pregabalin, twice a day - but actually my facial pain doesn't bother me anymore, unless I've been sleeping badly for a couple of nights or clenching too much. I don't have outliers anymore. The edges of my jaw are now the pain points. When I massage them, I get a toothache. One tooth in the back left has to come out again. It's as dead as a doornail but it hurts like hell the moment I get jaw trouble. I still do relaxation exercises on a regular basis. Just a few basics, to reduce any stimulus, because I get restless from too many stimuli. Looking back, it all makes sense.'**[6]**

Satisfied person

'I think it helped 90% and that the pain was reduced by at least 95%. There's a very satisfied person here.'**[7]**

She's still on medication. "I'm still taking two doses of 200 milligrams of Carbamazepine now. The last time I came off it, I didn't get a facial pain but a sort of stabbing nerve pain in my side, continuously on the left side. When I raised the dosage again to almost two, it was gone. So I'm now in the phase that I might have to take this my whole life. I'd like to discuss this further with my GP or somebody else. It would be nice to get rid of it once and for all but I am not ready for that step yet. I like the fact that the facial pain is now relatively zero and that means a lot to me after twenty years!'**[7]**

**Participant 8:** 'From two hundred percent shit to thirty, forty percent!’ It comes in spurts. 'I've had my best day ever two or three times now, so that's super good. But it'll go to extremes. The annoying thing is that you think it's stress related but it also happens that you're calmly thinking about what you're going to do during your day off and end up with mega pain in your mouth. Guys what?'**[8]**

**Participant 8** never took any pills. 'I can take whatever I want, but that pain in my mouth won't go away. Occasionally, I’ll use paracetamol and tea from Chinese acupuncturist.'**[8]**

Up and down

**Participant 9**: 'The pain has been reduced by at least 70%. I don't have any outliers either, but every now and then I feel pain in the morning. It's as if I may have had the wrong dream, or whatever. Maybe that's when I've been clenching my jaws together. That's why I prefer to make my appointments in the afternoons. I always need some time in the morning to calm down. I then notice it settling down. Because I have quite a bit of trouble and pain when eating. There are also periods that I can deal well with it, but at the moment I'm in an unpleasant situation again. Then I think: okay, it will pass. So it goes up and down. **'[9]**

**Participant 9** is in a phase of coming off their medication. 'I'm now only taking two pills at a time instead of five, so 2 × 100 milligrams.'**[9]**

Improved quality of life

'I was hoping, of course, to get rid of the pain... Well, not now,' says **Participant 10**. 'What has been built up in certain behaviour and physical symptoms over almost 55 years doesn't suddenly disappear in a year and a half. It's a very ingrained, powerful structure. There may be old behaviour, the causation may have disappeared, it may have dissolved, but its expression, or rather its behaviour, remains the same and that does not disappear. I also learned from the psychologist that pain tolerance decreases with age, which means sensitivity increases. So the pain may be objectively reduced, but not emotionally,'**[10]** **Participant 10** does not know what he can do to reduce the pain anyway. ‘For me, it has also become a way of life. That's why I hate it every time another tooth dies.’

He has stopped medication. 'A sleeping pill helps, so do narcotic antidepressants, but I find that awful. A tough and pioneering exercise right up to the pain threshold. That helps, for an hour or two.'**[10]**
'Access has been created to the largest source of tension: that sexual abuse, **Participant 10** continues. Very healing and very good, also very painful to do. But my mood swings and slightly depressed feelings have disappeared. I don't have the feeling anymore that sometimes I'm walking through mud and have to drag myself along. Life is literally more light-footed. In that respect, a burden has fallen off my shoulders and I feel more confident in myself, more liberated. I also like myself more, I'm nicer, so the quality has improved in a lot of ways.'**[10]**

Streamers:

*'This morning, I happened to be thinking about it. I was lying on my side, my whole nose was nicely open and I could therefore breathe easily... done!'*

*'In most cases, the pain has been reduced by as much as 60 to 70%’*

*‘During a busy working day, it can shoot back to 100%. But I'm less concerned about it'*

*'I don't think I'll ever get rid of it completely, but if I can do it this way, I'm actually completely satisfied'*

*'I still take medication - pregabalin, twice a day - but actually my facial pain doesn't bother me anymore’*

*'I think it helped 90% and that the pain was reduced by at least 95%. There's a very satisfied person here'*

*'From two hundred percent shit to thirty, forty percent!’*

*'The pain has been reduced by at least 70%'*

*'What has been built up in certain behaviour and physical symptoms over almost 55 years doesn't suddenly disappear in a year and a half. (...) The pain hasn't diminished but my quality of life has improved in a lot of ways'*

A message to the dental and medical sector

There are only ten of them, but they have each gained a lot of experience in their quest to be free of their pain. Do they have any recommendations within the medical sector?

**Participant 10:** 'If you look at the complexity and diversity of the complaints and the pains, the practitioners are apparently unable to say that it is tooth grinding. That relationship is not easily established. The advice is to arrive at a good management of the pain. But take a multidisciplinary approach, from different disciplines. So involve an ENT doctor, refer to a neurologist more quickly or appoint a case manager.'**[10]**

**Participant 1** makes a similar appeal. 'Many more general practitioners and dentists need to know about this, even ENT doctors. They sent me through the scan: "everything's fine...” I have advised several people in my area to go to the gnathologist. And if they ask me whether you should go to the psychologist, I'll talk openly about the importance of that.'**[1]**

**Participant 3:** 'A dentist needs to know about tongue pressing and what consequences this can have for facial pain. I see it almost as the basis. Look how many people are ultimately affected by it, including therapists themselves... So it's something that actually occurs much more often than people know. I really hope it starts to get more attention. I've dealt with this for three years, and I didn't have to.'**[3]**

Make referrals!

**Participant 6:** 'I think the GP should have taken other steps first. To what extent do you have contact about this?'**[6]**

**Participant 8:** 'I have called back a number of therapists: if something like this ever happens again, for God's sake, refer them to a gnathologist. Because that's where you'll find someone who at least recognises the symptoms.'**[8]**

In hindsight, **participant 9** thinks she was hurt by those Sweet procedures. 'My anaesthesiologist also told me that you retain pain after a third or fourth time. Even though I've had two treatments, I think it's the same for me. I would have been better off with that Jannetta surgery right away. I think it's a pity that GPs and pain specialists don't refer someone with facial pain directly to a TMD team. Maybe they know, but they don't gravitate towards it. Or maybe they're not allowed to? A pain specialist needs to know how to refer a patient. That should be done nationwide.'**[9]**

Preferably non-invasive

'You always think that an operation is the best solution right away, that it will solve the problem,' says **Participant 2**. 'But my surgery was very difficult. It took me a long time to recover and since then I can't work regularly, I can't get it into my head anymore. When I then see how the TMD work ultimately had such a great affect! So maybe that operation wasn't necessary.'**[2]**

**Participant 2** gives another example: 'While waiting to get to see the TMD team, a neurologist, according to his protocol, had me try five medications. Both processes are very long. That's why it would have been nice if the TMD team had looked at those medications to see if something else was going on. Of course there was the underlying TMD case that also caused the symptoms.'**[2]**

Look over the wall

**Participant 5:** 'First enlist a TMD team before any operation. I think that facial pain is often related to the jaw, but it was never looked at, at least not in my case. If I had been referred earlier, it would have saved me a lot of severe pain, at least it would have been worth a try. I was taking so much medication that I actually couldn't function. And in hindsight I don't think I should have had the surgery.'**[5]**

It would be nice if every doctor looked over his own wall, beyond his own discipline. And that there is more collaboration between the different fields, **Participant 7** concludes. 'That's what I like about the three-pronged TMD process. It would be nice if all the knowledge there is about facial pain or TMD were communicated to all sector associations. And that the practitioners, the dentist and the like, can immediately recognise those symptoms and take it seriously. That you can then walk the right path. I don't know if all patients would benefit from that. But it's much nicer to do something non-invasive. The recovery process of an operation is much harder.'**[7]**

Streamers:

*'If you look at the complexity and diversity of the complaints and the pains, the practitioners are apparently unable to say that it is tooth'*

*'Many more general practitioners and dentists need to know about this, even ENT doctors'*

*'I have called back a number of therapists: if something like this ever happens again, for God's sake, refer them to a gnathologist'*

*‘I'd like it if a lot of dentists knew about this’*

*‘If I had been referred sooner, it would have saved me a lot of severe pain’*

*‘It would be nice if every doctor looked over his own wall, beyond his own'*

*‘But it's much nicer to do something non-invasive; the recovery process of an operation is much harder.’*

Message to a multidisciplinary team

How do the participants look back on their experiences with the TMD team? Are there any tips?

**Participant 1:** 'I've been incredibly satisfied with this process. The only thing: I had to go to Boxmeer, Groesbeek and Nijmegen. It would be a lot easier if the team was in one place.'**[1]**

Also **Participant 3** was very satisfied. 'The type of people, all their own sub-disciplines... I've learned a lot from them.'**[3]**

**Participant 6:** 'Because I didn't really think the team could solve my problem, it was tricky. I looked for help elsewhere. In that sense, I always wondered: to what extent is there contact with the GP about this? Zero? Inter-collegial consultation might have convinced me. If the GP had said to me: "Listen, you went to the first-line psychologist and nothing came out of it... I still have one more route open for you, and I'd recommend that...” I guess I would have said: "Okay, if that's what you say, the dentist said it too...” Then you get the combination that might have been a step to convince me to start with that.'**[6]**

Follow-up

An osteopath could be a very useful addition to the team, thinks **Participant 8**. 'Because it's mainly about teaching your body to walk a different path. A piece of relaxation that you eventually have to do yourself. Could you link something like that to EMDR?'**[8]**

**Participant 9:** ‘I don't think you have anything to improve.’**[9]**

**Participant 10:** 'There should be a follow-up when it comes to pain management. If, for example, trigger point massage doesn't work, an alternative should be sought. Within the TMD process, we are constantly fixed on the causation, the causality, but the pain doesn't go away. That was the reason I came, with all its side effects and misery and complaints that limit the quality of my life. Isn't there something more effective against that pain? Every now and then I think: I'll pull all the teeth out of my jaws, get a rubber denture and then I'll be rid of it...'**[10]**

Streamers:

*'It would be a lot easier if the team was in one place'*

*'An osteopath could be a very useful addition to the team. Because it's mainly about teaching your body to walk a different path'*

*'Within the TMD process, we are constantly fixed on the causation, the causality, but the pain doesn't go away. (…) Every now and then I think: I'll pull all the teeth out of my jaws, get a rubber denture and then I'll be rid of it...'*
